# Polyploid giant cancer cells (PGCC): short-term return to multicellularity

**DOI:** 10.1186/s40659-025-00650-1

**Published:** 2025-11-24

**Authors:** Alexander E. Vinogradov, Olga V. Anatskaya

**Affiliations:** 1 Institute of Cytology, Russian Academy of Sciences, St. Petersburg, 194064 Russia; 2 Center of Genetic Reprogramming and Gene Therapy, Institute of Cytology RAS, St. Petersburg, Russia

**Keywords:** Atavistic reversal, Ontogenetic reversal, Transcriptomes, Gene expression, Gene phylostratigraphy, Cancer, Oncogenesis, Stemness, Polyploidy, Evolutionary medicine

## Abstract

**Background:**

Polyploidization is associated with progression of cancer, making cancer cells more dangerous. The common polyploid cancer cells constitute a considerable part of tumors (up to 56% in metastases). The giant polyploid cancer cells (PGCC), which appear under severe stress caused by treatment when the majority of cells die, present an enigmatic phenomenon both in fundamental and practical sense because they develop treatment resistance.

**Results:**

Using transcriptome meta-analysis, we studied different types of polyploid cancer cells and found that in common polyploid cancer cells, the genes of unicellular (UC) origin and stemness are upregulated (compared to diploid cancer cells). At that, the upregulated UC genes show a higher local and global protein interactome centrality than the upregulated stemness genes, suggesting that the UC interactome attractor is a driving force behind this backward movement along the evodevo axis. Surprisingly, PGCC show the opposite picture. There occurs the suppression of UC and stemness genes with the upregulation of multicellular genes (especially those involved in intercellular communication), suggesting a reversal towards multicellular (MC) state. This effect is enhanced in PGCC's early progeny but diminished in the late progeny, indicating its transient nature. PGCC of different origin (breast, ovarian, prostate cancers), induced by different stresses (radiation or drugs with various mechanisms of action), show a similar behavior. The first principal component of transcriptome profiles, which is common for all cell types (initial cancer cells, PGCC, early and late progeny) and contains the major part of expression variance, is also directed along the gene evolutionary age axis.

**Conclusions:**

While the common polyploid cancer cells comply with the 'serial atavism' model of oncogenesis, PGCC present a unique phenomenon of the short-term return to multicellularity probably associated with collective acquisition of resistance to treatment. Our analysis revealed also the evolutionary origin of the main differences in gene expression, emphasizing the importance of gene age axis in transcriptome analyses. The deep evolutionary basis of variation in gene expression across and within cell types might become a general framework for interrelated problems of cell and cancer biology and regenerative medicine.

**Supplementary Information:**

The online version contains supplementary material available at 10.1186/s40659-025-00650-1.

## Introduction

Notwithstanding the War on Cancer declared in 1971, the burden of cancer is growing in the whole world [1]. While death from some cancers is reducing, the other cancer types show an increased mortality [1, 2]. The global burden is expected to rise by 47% from 2020 to 2040 [3]. Recently, the early-onset cancer was considered an emerging global epidemic [4]. The other great problem is the appearance of cancer cells resistant to treatment [5–8]. Still other impetus to this field is associated with therapeutic regeneration of damaged tissues, which may lead to oncogenesis [9–12]. This complexity of the whole knot of interrelated problems suggests the growing need in prevention, early detection, and treatment efforts, which might be based not only on the empirical ground but on the more profound understanding of cancer nature. We suppose that this understanding can be achieved by analysis of cell movement along the evodevo (unicellularity/stemness) axis, which is a consistent and comparable characteristic having the deep evolutionary origin. Therefore it might become a general framework for the interrelated problems of cancer biology and regenerative medicine.

The cancer hallmarks initially proposed by Hanahan and Weinberg and later expanded upon, include proliferative advantage, replicative immortality, inducing/accessing of blood vessels, invasion and metastasis, reprogramming cellular metabolism, evading immune destruction and apoptosis, dedifferentiation, and transdifferentiation [13–17]. Albeit these hallmarks provide a fine description of what is altered in cancer cells, they do not explain why these changes appeared (i.e., the causes and mechanisms of oncogenesis) [16]. Recently, the reversal to a unicellular (UC)-like state was proposed as a universal hallmark, which is based on the evolutionarily-formed mechanism (UC attractor of cellular networks) and can explain both the other hallmarks and the causes of cancer [18, 19]. However, this proposal was complicated by the fundamental problem. Because of the biogenetic (recapitulation) law, ontogenesis partially recapitulates phylogenesis even at the cellular level [20, 21], which prevented distinction between the evolutionary (atavism) and ontogenetic (dedifferentiation) reversals. Yet, recently it has been shown that while the ontogenetic signature is stronger in prediction of normal stem vs. differentiated cell state than the UC signature, the UC signature is stronger in prediction of cancer vs. normal cell state [22]. This observation justifies the atavistic reversal as a central hallmark of cancer. In a practical sense, it means that i) predominance of UC signature over stemness signature suggests an oncogenic threat, and ii) for safe regenerative medicine, it is necessary to achieve the ontogenetic reversal to stemness without the atavistic reversal to a UC-like state.

Polyploidization is associated with progression of cancer, making cancer cells more dangerous [23]. The common (ordinary) polyploid cells, which arise because of disruption of mitosis or cytokinesis or via cell fusion, constitute up to 56% in metastatic solid tumors [24]. Much rarer giant polyploid cancer cells (PGCC) are more enigmatic. They arise in response to severe sublethal stress (usually caused by cancer treatment) when the most cells die [25–27]. PGCC stop division and after certain time can proliferate by amitosis producing near-diploid progeny, which become resistant to a stressful factor [25–27]. Due to their unusual type of proliferation and acquisition of resistance to treatment, PGCC present an enigmatic biological and clinical phenomenon (first of all, in regard to possible heritage from their ontogenetic or phylogenetic past, which they use).

Here we used the transcriptome meta-analysis to investigate the movement along the evodevo axis (i.e., changes in the expression of ontogenetic/stemness and phylogenetic/unicellular gene signatures) in common polyploid cancer cells and in PGCC. We found a sharp contrast between them. The common polyploid cancer cells show an enhancement of the UC-biased reversal probably caused by the UC attractor of cellular networks. On the contrary, PGCC show the opposite movement towards multicellularity.

## Methods

### Transcriptomes of polyploid cancer cells

The data on the genes, which are differentially expressed in common (ordinary) polyploid vs. diploid cancer cells, were acquired from the work [23]. They contained the polyploid/diploid folds for various cancers. The 'pancancer' data (integrated over about 10,000 cancer samples) were used.

The transcriptome databases of polyploid giant cancer cells (PGCC) were acquired from Gene Expression Omnibus (GEO) (https://www.ncbi.nlm.nih.gov/gds). For an exemplar PGCC analysis, we selected the transcriptomes of prostate cancer cell line (PPC1), where PGCC were obtained by radiation (GSE196453) [28], because it is the most complete dataset containing the initial diploid cells, PGCC, their early progeny (8 day) and the latest available (20 day) progeny, with the highest numbers of replicates and genes. To check the generality of revealed effects, we studied also other available PGCC databases. The first additional database is the prostate cancer cell line (PPC1) with radiation-induced PGCC, treated or non-treated with pro-drug LCL521, which is an inhibitor of lysosomal enzyme acid ceramidase ASAH1 (GSE195919) [29]. The second additional database contains the two ovarian cancers cell lines (Hey and SKOV3), where PGCC were induced by treatment with tubulin-targeting drug paclitaxel (GSE178745) [30]. The third additional database contains four permanent cell lines from ovarian and breast cancers (Hey, MCF7, OVCA432, SKOV3) and three primary cell lines (Org2414, Org2445, Org3008), obtained from individual human high-grade serous ovarian cancers and cultivated in patient-derived xenografts (GSE229119) [31]. PGCC were induced by treatment with olaparib, which is an inhibitor of poly ADP ribose polymerase (PARP). At last, the fourth additional database contains three breast cancer cell lines (MDA, SUM159, Vari068), where PGCC were induced by treatment with cytoskeletal drug docetaxel (GSE248717) [32]. We used the processed data and made comparisons pairwisely only within the datasets obtained by the same method in the same laboratory. Summary information on the PGCC transcriptome databases is shown in Supplementary Table 1.

### Gene signatures

The evolutionary stratification (phylostratigraphy, or gene dating) of human genes was acquired from the work [33], where the problem of different gene datings was discussed, and the shallow vs. deep phylostratigraphies were introduced. Here, we used mostly the shallow phylostratigraphy, which is based on the strict gene orthology obtained using best reciprocal hits with the Smith–Waterman algorithm (which is more accurate than BLAST). The phylostrata are shown in the corresponding figure legends. (The phylostrata labels are according to NCBI taxonomy.) The deep phylostratigraphy (which includes in-paralogous genes), providing dating of whole gene families, was used as an additional parameter. It is presented by the multicellularity gene index (MGI), which indicates how typical is a gene for MC organisms [33]. If MGI is equal to unity, the gene family was found only in MC organisms. If MGI is below unity, the gene family has a deep UC origin. The Transcriptome Age Index (TAI) was calculated according to the work [34].

The stemness signatures were acquired from the work [35], which is a comprehensive compendium of many stem cell databases. Each gene is characterized by the 'Multi' score indicating the number of multipotent cell databases, where a gene was found, and the 'Pluri' score indicating the number of pluripotent cell databases, where a gene was found. The 'Stem' score indicates the integrated 'Multi' and 'Pluri' scores. The zero score means non-stemness. The list of well-recognized oncofetal genes was acquired from the work [36].

The other gene signatures were acquired from Gene Ontology (GO). For each GO category, we collected all its subcategories using GO directed acyclic graphs (DAG), and a gene was regarded as belonging to a given category if it was mapped to any of its subcategories. This is necessary because many genes are mapped only to their specific categories and not to more general categories. We used the following GO categories: 'nucleus' (GO:0005634), 'cytoplasm' (GO:0005737), 'plasma membrane' (GO:0005886), 'signaling receptor activity' (GO:0038023), 'channel activity' (GO:0015267), 'gap junction' (GO:0005921), and 'mitotic cell cycle' (GO:0000278).

### Protein interactions

The human pairwise protein interactions were acquired from STRING database [37]. We selected the interactions with a top-half confidence (> 0.5), which is higher than default confidence used by STRING server (> 0.4). The measures of protein interactome centrality (local 'degree' and global 'betweenness' and 'stress') for each protein were determined using Cytoscape (version 3.10.1) [38]. They were used for analysis of common polyploid cancer cells.

To verify the results of the whole transcriptome analysis of PGCC, we studied the strongly up- and downregulated protein interaction hubs (> 5 interactants, interaction confidence > 0.7, expression fold > twofold) in PGCC compared with initial cancer cells. They were clustered by K-means algorithm and analyzed to identify biologically meaningful modules using STRING server.

### Statistical analyses

We mapped gene symbols to NCBI identifiers. For better comparability of transcriptomes and gene signatures, we standardized the data (i.e., expressed them in standard deviations with mean as zero, thus removing possible differences in means and variances), as previously [22]. In most analyzes, there was also an independent internal normalization for revealing the direction of cell movement along the evodevo axis: the certain signatures were upregulated, whereas their complementaries were downregulated (e.g., UC vs. MC, stemness vs. non-stemness). In the case of comparison of individual oncofetal genes we used pair-wise whole-transcriptome normalization by mean to preserve original expression levels. The main statistical analysis was ANOM (analysis of means) [39]. The analyses and visualization were performed using Statgraphics Centurion, Prism, R package, Cytoscape, and STRING server.

## Results

### Common polyploid cancer cells

**Fig. 1:**
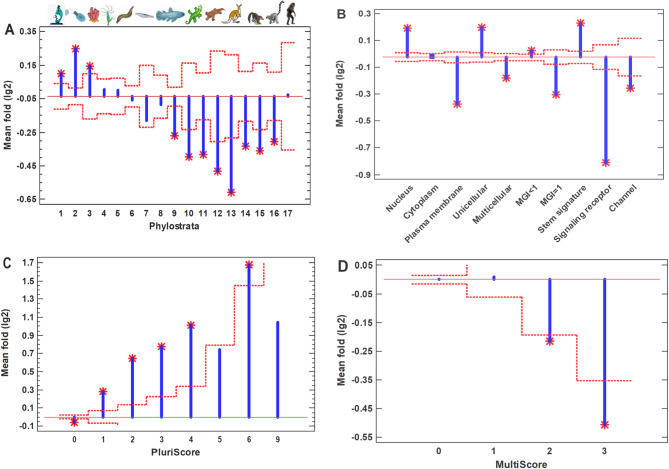
Characteristics of common (ordinary) polyploid cancer cells, compared to diploid cancer cells. **A** – Evolutionary profile of gene expression fold. Phylostrata: 1—cellular organisms (Prokaryota); 2—Eukaryota; 3—Opisthokonta; 4—Metazoa; 5—Eumetazoa; 6—Bilateria; 7—Chordata; 8—Vertebrata; 9—Euteleostomi; 10—Tetrapoda; 11—Amniota; 12—Mammalia; 13—Theria; 14—Eutheria; 15—Boreoeutheria; 16—Primates; 17—Hominidae. (First three phylostrata are unicellular. The pictures at the top show recent organisms corresponding to phyletic branching used for human gene dating.) **B** – Gene expression folds for different signatures (MGI, multicellularity gene index). **C** – Gene expression folds for pluripotent genes (PluriScore, the number of pluripotent cell databases, where a gene is present). **D** – Gene expression folds for multipotent genes (MultiScore, the number of multipotent cell databases, where a gene is present. Red dotted lines show confidence intervals (*p* = 0.05), red stars – significant differences

We studied the common polyploid cancer cells using the collective data obtained from the integration of about ten thousands samples ('pancancer' dataset) [23]. Usually, cancer tissues contain up to 56% of polyploid cells but the polyploid giant cancer cells (PGCC), which are studied in the next section, can present only a small fraction in usual cancer samples [26–28]. Therefore, we assumed that the 'pancancer' dataset contains mostly the common (non-giant) polyploid cancer cells. Compared to diploid cancer cells, the common polyploid cancer cells show the upregulation of UC-origin genes with the suppression of MC genes (Fig. 1A). Notably, this three-phase evolutionary profile (activated UC phylostrata, roughly non-changed middle phylostrata, and suppressed late MC phylostrata) strikingly resembles the saturated pattern of random-walk modeling in the human interactome [19]. This pattern is presumably the result of UC attractor arising because of a higher local and global protein interaction density in the ancient UC center (which, in turn, is the result of the core-to-periphery evolutionary growth of interactome) [19]. This effect completely remains after the removing of cell cycle genes from analysis (Supplementary Fig. 1A). Therefore, it cannot be explained by possible differences in cell proliferation status.

The backward movement along the evodevo axis is manifested not only in the upregulation of strictly UC genes (determined using the 'shallow' phylostratigraphy) but also by the deep gene dating of gene families. (This two types of gene dating were introduced and discussed in the work [33].) Thus, the genes with the multicellularity gene index (MGI) below unity (their families are of deep UC origin) were upregulated, whereas the genes, whose MGI is equal to unity (MC gene families), were suppressed (Fig. 1B). Also, the enhanced expression was observed for the nucleus-mapped and stemness genes, whereas the genes belonging to the plasma membrane, signaling receptors, and membrane channels were downregulated (Fig. 1B). Again, these effects completely remain after removing of cell cycle genes from analysis (Supplementary Fig. 1B). Because of the cellular biogenetic law [21], the UC and stemness genes show a similar behavior. Notably, among the stemness genes, the upregulation is seen mostly in the pluripotent genes, whereas the multipotent genes are rather downregulated (Fig. 1C, D; Supplementary Fig. 1C,D). This observation indicates the activation of the 'deep' stemness in common polyploid cancer cells (compared to diploid cancer cells).

**Fig. 2:**
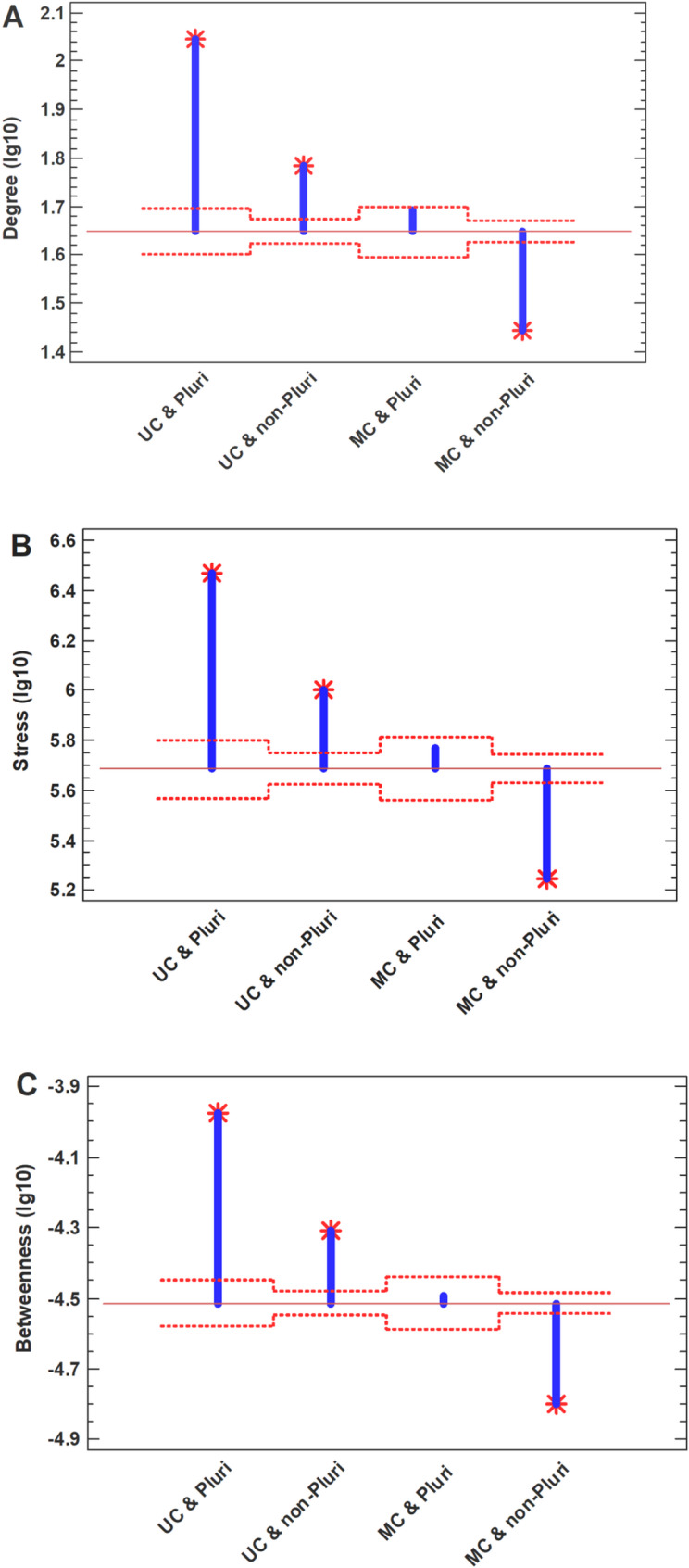
Protein interactome centrality measures for the upregulated genes in common polyploid cancer cells (compared to diploid cancer cells).** A** – Degree, the number of direct (one-step) interactions of a given protein (local centrality measure). **B** – Stress, the total number of shortest paths between all pairs of other proteins passing through a given protein (global centrality measure showing load or traffic on a network node). **C** – Betweenness, similar to stress but paths are weighted by inverse of total paths (global centrality measure showing control or brokerage role of a node in the network). Red dotted lines show confidence intervals (*p* = 0.05), red stars – significant differences

To disentangle the evodevo axis, we analyzed the local and global protein interactome centrality for the upregulated UC and pluripotency genes. The highest centrality was observed for the genes belonging both to UC origin and pluripotency, the second place was for UC but non-pluripotent genes, the third, for pluripotent but non-UC genes, and the fourth, for non-pluripotent non-UC genes (Fig. 2A–C). The effect remains (and even enhances) after removing of cell cycle genes from analysis (Supplementary Fig. 2A-C). These findings suggest that it is the UC-origin genes, presumably forming the UC interactome attractor [19], which are the driving force behind the backward movement of polyploid cancer cell along the evodevo axis. This is in agreement with the recent report that the UC signature is a stronger predictor of cancer than the stemness signature in the cancer vs. normal cells [22]. Considering polyploidization of cancer cells as progression of cancer [23], this observation agrees with the 'serial atavism' model suggesting that cancer onset and progression involve a series of evolutionary reversals [40].

### Polyploid giant cancer cells (PGCC)

For an exemplar analysis of polyploid giant cancer cells (PGCC), we selected the transcriptomes of prostate cancer cell line (PPC1), where PGCC were obtained by radiation [28]. It is the most complete dataset containing the transcriptomes of initial diploid cells, PGCC, their early (8 day) progeny, and the latest available (20 day) progeny, with the highest number of replicates and genes. The analysis of other databases supporting the generality of the observed effects is described in the next section.

**Fig. 3:**
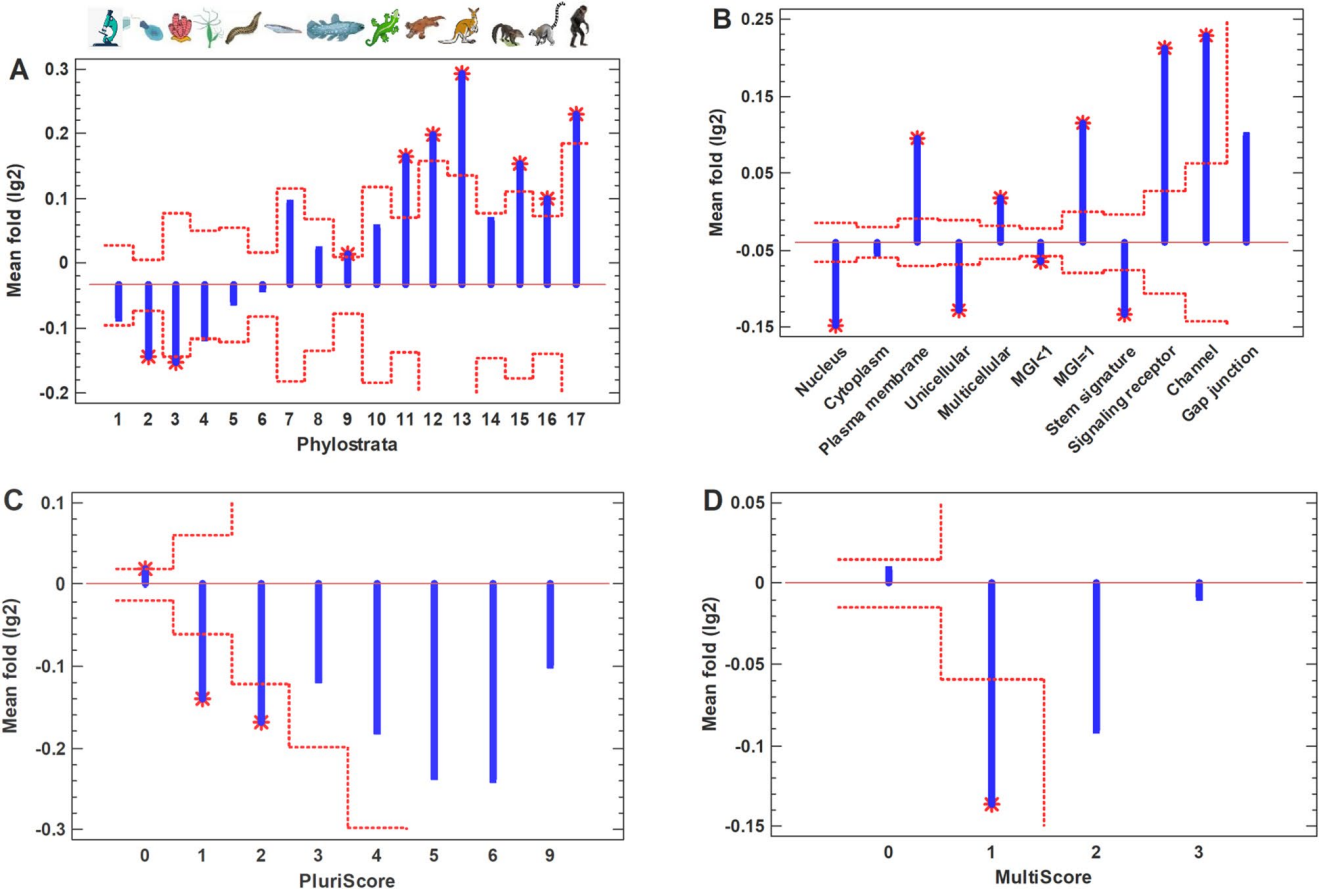
Characteristics of PGCC, compared to initial cancer cells (from GSE196453). **A** -- Evolutionary profile of gene expression fold. Phylostrata: 1—cellular organisms (Prokaryota); 2—Eukaryota; 3—Opisthokonta; 4—Metazoa; 5—Eumetazoa; 6—Bilateria; 7—Chordata; 8—Vertebrata; 9—Euteleostomi; 10—Tetrapoda; 11—Amniota; 12—Mammalia; 13—Theria; 14—Eutheria; 15—Boreoeutheria; 16—Primates; 17—Hominidae. (First three phylostrata are unicellular. The pictures at the top show recent organisms corresponding to phyletic branching used for human gene dating.) **B** – Gene expression folds for different signatures (MGI, multicellularity gene index). **C** -- Gene expression folds for pluripotent genes (PluriScore, the number of pluripotent cell databases, where a gene is present). **D** -- Gene expression folds for multipotent genes (MultiScore, the number of multipotent cell databases, where a gene is present). Red dotted lines show confidence intervals (p=0.05), red stars – significant differences.

**Fig. 4:**
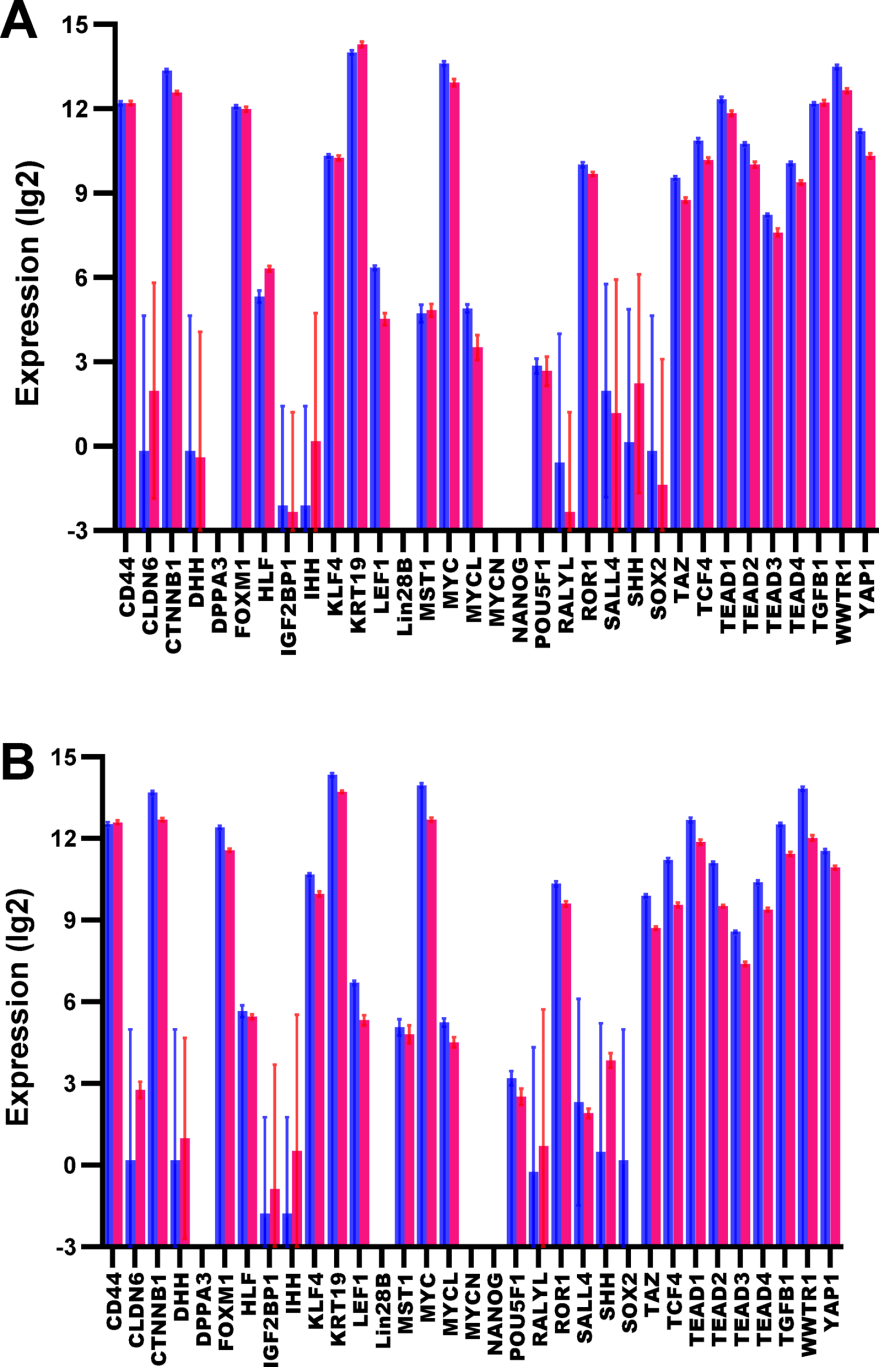
Expression levels of the oncofetal genes in tested cells (red), compared to initial cancer cells (blue). **A** – PGCC; **B** – PGCC's early progeny

Surprisingly, in contrast to the common polyploid cancer cells, the PGCC show the opposite movement along the evodevo axis (compared to diploid cancer cells, from which they were obtained). They shifted to multicellularity, with the upregulation of MC genes, plasma membrane genes, signaling receptors, and membrane channels, and the suppression of UC genes, nucleus-mapped and stemness genes (Fig. 3A, B; Supplementary Fig. 3A, B). This is a counterintuitive effect not only because the common polyploid cancer cells show the movement in the opposite direction but also taking into account the morphology and propagation mode (amitosis) of PGCC, which are similar to certain ancient (UC) and/or embryonic (because of the biogenetic law) patterns [41, 42]. Therefore, in addition to the general evodevo (unicellularity and stemness) signatures, we analyzed the changes in the well-recognized oncofetal genes that are typical for cancer stem cells [36], taken separately. In PGCC, only two oncofetal genes (HLF and KRT19) showed a statistically significant upregulation, whereas 13 genes (CTNB1, LEF1, MYC, MYCL, RORF1, TAZ, TCF4, TEAD1-4, WWTR1, YAP1) were downregulated (Fig. 4A). (Totally, there were 33 genes but the other genes were not changed significantly.)

**Fig. 5:**
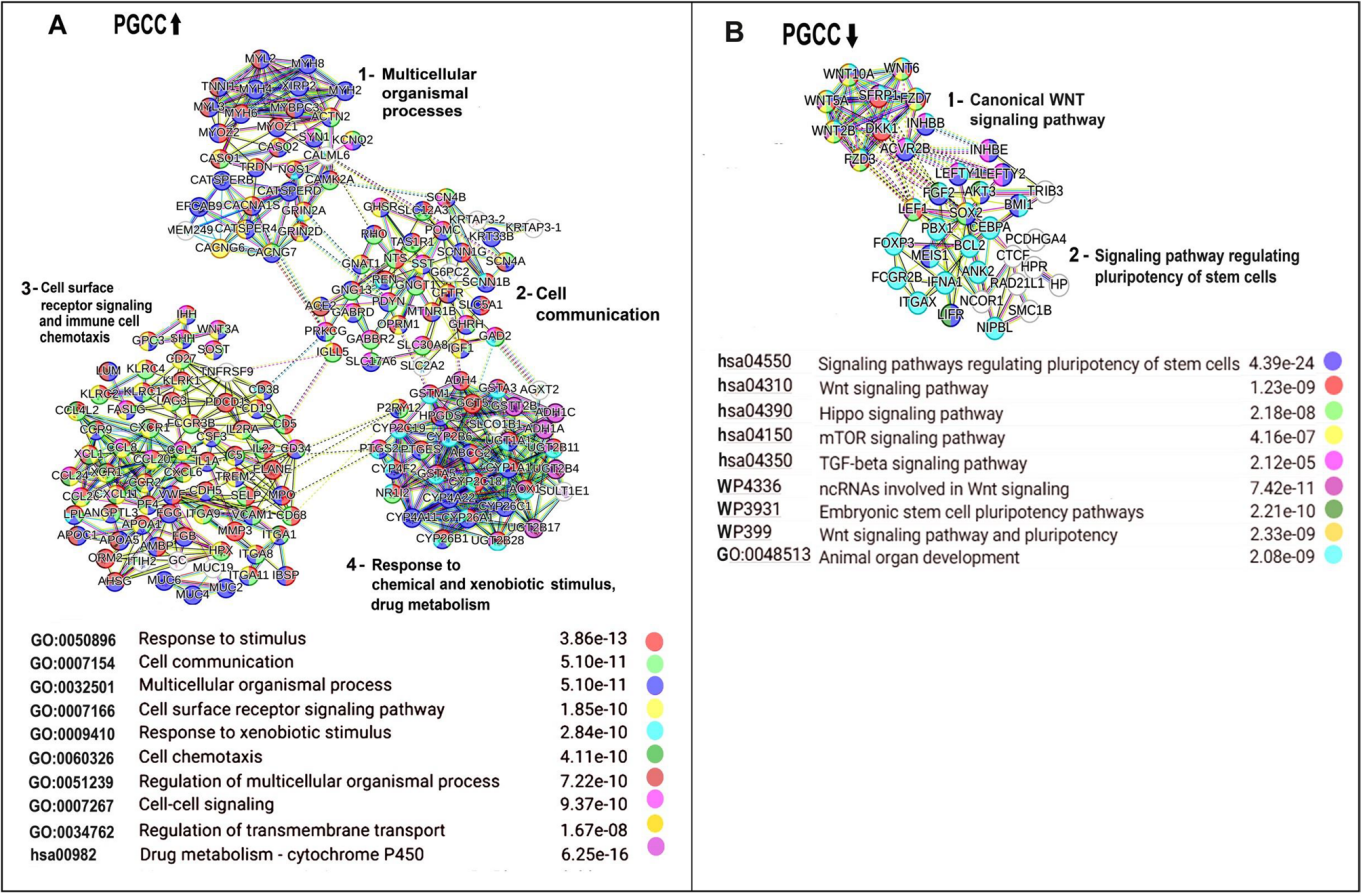
Protein interactome clusters in the upregulated (**A**) and downregulated (**B**) hubs of PGCC, compared to initial cancer cells

To verify the results of the whole transcriptome analysis, we studied also the strong protein interactome hubs, which were up- or downregulated in PGCC compared with initial cancer cells. They were clustered using K-means algorithm to identify interactome modules (Fig. 5). The results were in good agreement with the transcriptome-wide analysis. The upregulated hubs involved multicellularity and intercellular communication seen in the three clusters. (1**) "**Multicellular organismal processes**" (**regulated by structural and motor proteins MYL, MYH, ACTN2, CASQ2). (2) "Cell communication" with G-protein coupled receptors (ADRBs, CHRM, GABBR2) and ion channels (SCNAs, KCNQs). (3) "Cell surface receptor signaling pathways and immune cell chemotaxis" with cytokine receptors (CCR, CXCR, ILRs), adhesion molecules (VCAM1, ICAMs), and MHC-related proteins pointing to the modulating of tumor immune microenvironment and immune evasion or recruitment of tumor-promoting leukocytes. In addition, there was the fourth cluster: (4) "Response to chemical and xenobiotic stimulus, drug metabolism", including the highly significant pathway "Drug metabolism–cytochrome p-450" (*p* < 6.25e−16) containing cytochrome P450 enzymes (CYP2C, CYP3A, CYP26, UGT, GSTs). This result showed that PGCC developed defense against chemotherapeutic agents, underlying their resistance to therapy. It should be emphasized that these PGCC were obtained by radiation. Therefore, the activation of chemical response indicates the emergence of a generalized stress-resistance.

In the downregulated hubs, there were the two clusters. (1) "Canonical WNT signaling" (including WNT10A, WNT6, WNT5A, FZD7, DKK1). These genes maintain stemness, self-renewal, and developmental plasticity. (2) "Signaling pathway regulating pluripotency of stem cells" is enriched in the components of pluripotency regulatory signaling network (such genes as LEFTY1/2, FGF2, AKT3, BMI1, PBX1, CTCF). This cluster reflects stem cell-like transcriptional regulation. These results indicated the downregulation of important developmental and stemness pathways.

**Fig. 6:**
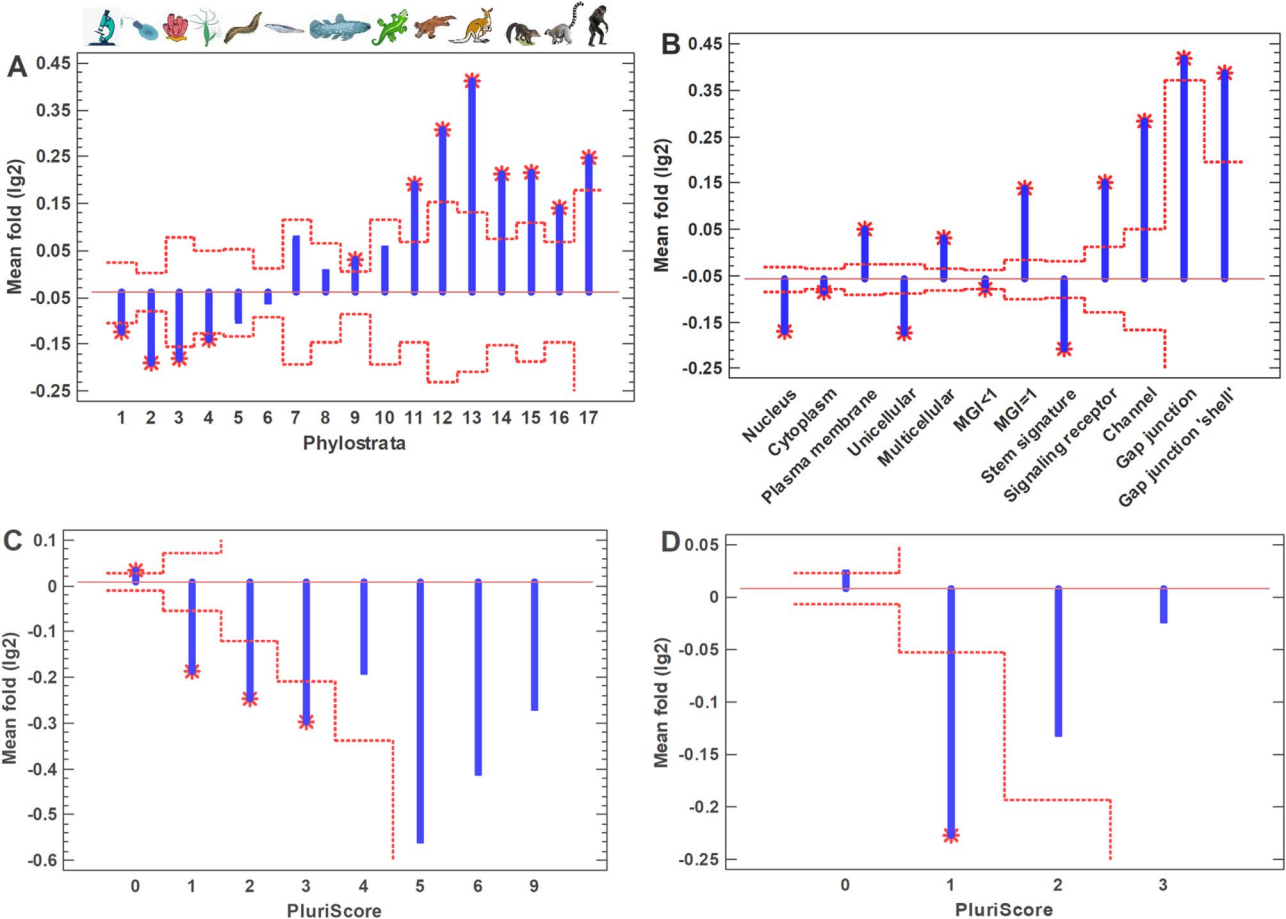
Characteristics of PGCC's early progeny, compared to initial diploid cancer cells (from GSE196453), with cell cycle genes excluded. **A** – Evolutionary profile of gene expression fold. Phylostrata: 1—cellular organisms (Prokaryota); 2—Eukaryota; 3—Opisthokonta; 4—Metazoa; 5—Eumetazoa; 6—Bilateria; 7—Chordata; 8—Vertebrata; 9—Euteleostomi; 10—Tetrapoda; 11—Amniota; 12—Mammalia; 13—Theria; 14—Eutheria; 15—Boreoeutheria; 16—Primates; 17—Hominidae. (First three phylostrata are unicellular. The pictures at the top show recent organisms corresponding to phyletic branching used for human gene dating.) **B** – Gene expression folds for different signatures (MGI, multicellularity gene index; Gap junction 'shell' (STRING's term for protein interactome neighborhood). **C** – Gene expression folds for pluripotent genes (PluriScore, the number of pluripotent cell databases, where a gene is present). **D** – Gene expression folds for multipotent genes (MultiScore, the number of multipotent cell databases, where a gene is present). Red dotted lines show confidence intervals (*p* = 0.05), red stars – significant differences

**Fig. 7:**
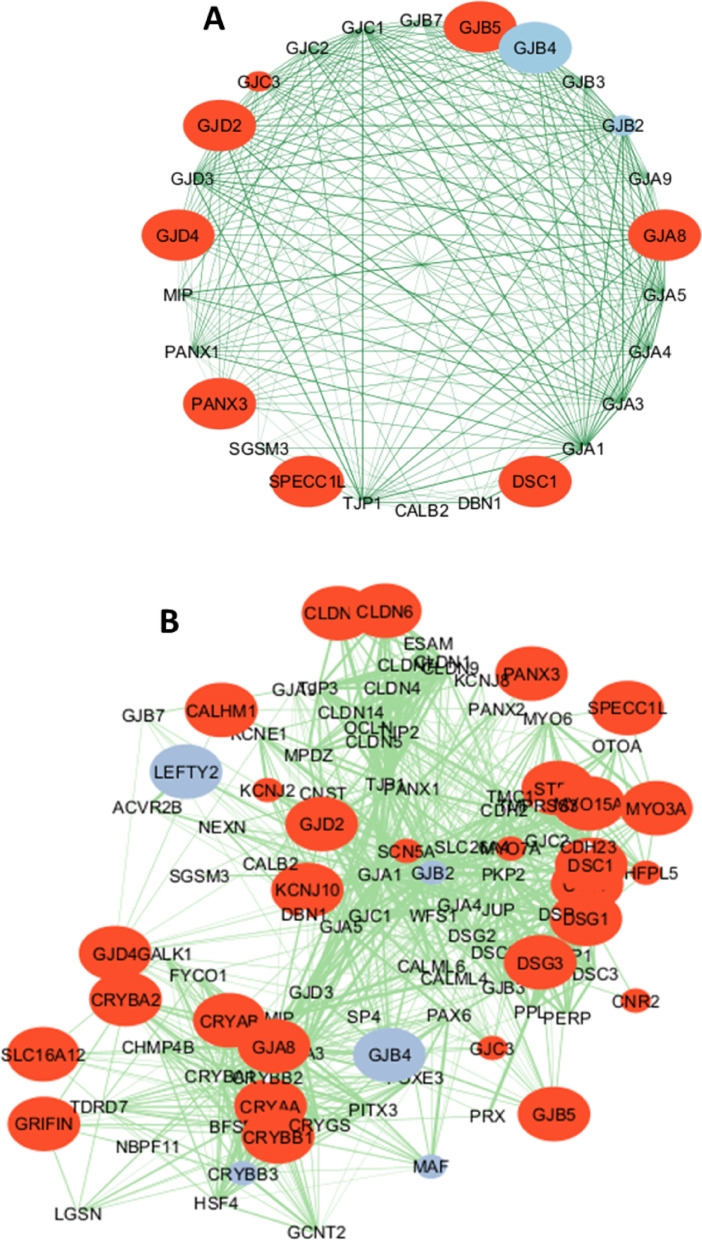
Protein interactome networks. **A** Gap junction proteins. **B** Gap junction proteins with their 'shell' (STRING's term for protein interactome neighborhood)**.** Small red circles, upregulated with fold > 2, large red circles, with fold > 4. Small blue circles, downregulated with fold > 2, large blue circles, with fold > 4. The width of lines correlates with confidence of interactions

The activation of multicellularity is more pronounced in PGCC's early progeny (Fig. 6; Supplementary Fig. 4). In addition, gap junctions became upregulated significantly (Fig. 6B). Using the principle of network propagation [43], we found that their protein interactome neighborhood is similarly activated (Fig. 6B). The analysis of gap junction network and its neighborhood showed that there are much more strongly upregulated than suppressed genes (Fig. 7). This network contains cell communication and sensory modules (Supplementary Table 2), which support the reversal to multicellularity. In tumorigenic aspect, of special interest are crystallins (CRYAA, CRYAB, CRYBB1). They are small heat shock proteins, which protect against oxidative stress and apoptosis in normal and cancer cells [44, 45]. CRYAB in particular is well-known to promote metastasis and therapy resistance; which can serve as a diagnostic marker and therapeutic target [46–48]. Claudin family genes (e.g., CLDN6, CLDN19) are also served as biomarkers and therapeutic targets across cancers [49]. The similar role is placed by certain ion channels (e.g., SCN5A) [50, 51]. No oncofetal genes were significantly upregulated in PGCC's early progeny, whereas 19 genes were suppressed (including HLF and KRT19) (Fig. 4B), compared with initial cancer cells.

**Fig. 8:**
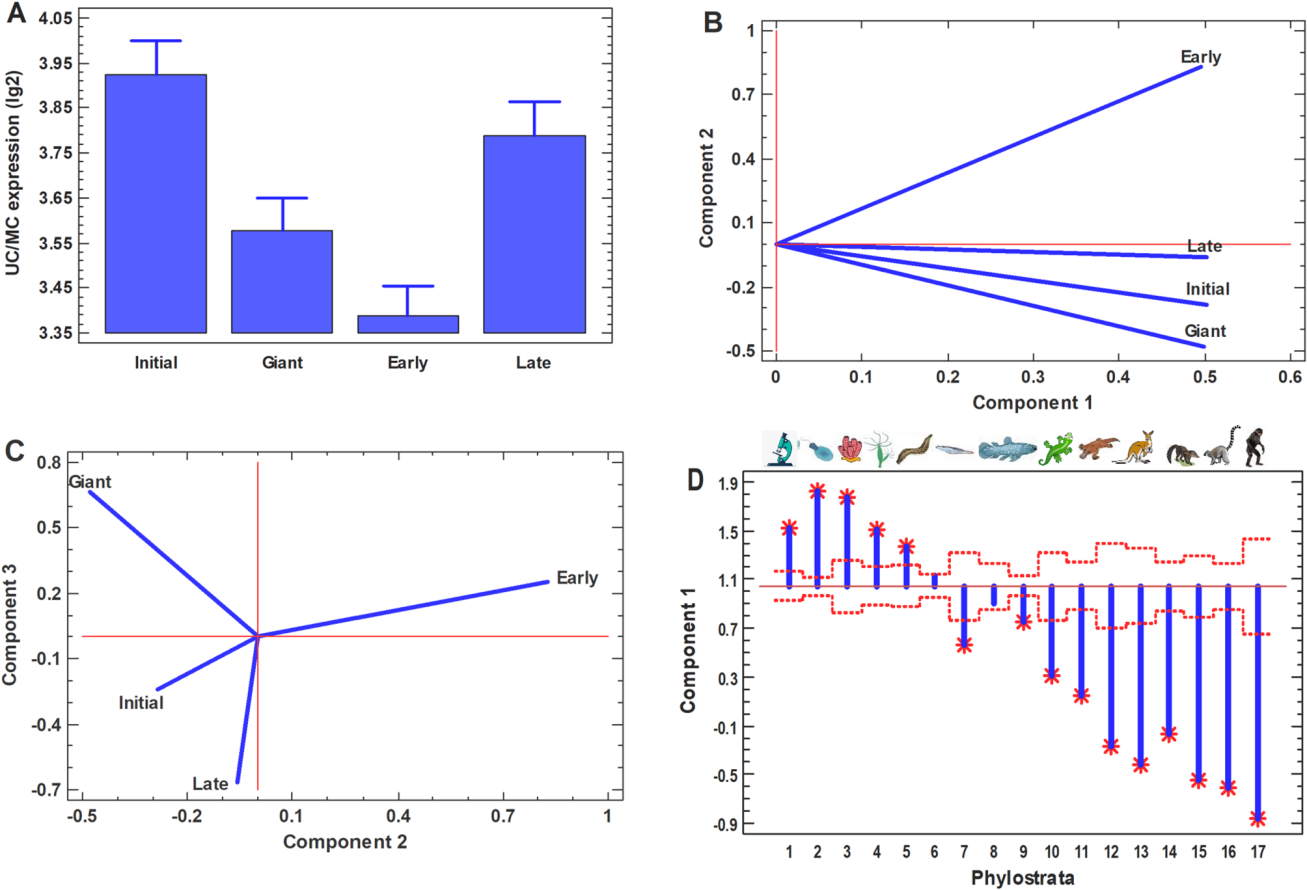
General picture for all four cell types (initial cancer cells, PGCC, their early and later progeny). **A** UC/MC gene expression ratio; **B**–**D** – principal component analysis (PCA) of the whole transcriptomes

In the late progeny, there was the backward movement to unicellularity (Supplementary Fig. 5A-D), albeit it did not reach the UC level observed in the initial cells (Supplementary Fig. 6A-D). The integrated picture for all four cell types (initial, PGCC, early and late progeny) is shown in Fig. 8 (Supplementary Fig. 7).

The principal component analysis (PCA) of whole transcriptomes for all four cell types showed that the first component is practically equal for all cell types (Fig. 8B; Supplementary Fig. 7B). The second and third components show that the early progeny is most distant from other cell types, whereas the late progeny is closest to initial cancer cells (Fig. 8C; Supplementary Fig. 7C), which is in agreement with the analysis of the UC signature (Fig. 8A; Supplementary Fig. 7A). The Transcriptome Age Index (TAI) also supports this conclusion (Supplementary Fig. 8).

We also found that the first principal component of transcriptome profiles, which is common for all studied cell types (initial cancer cells, PGCC, the early and late progeny) and contains the major part (above 90%) of total gene expression variance, is correlated with the gene age (Fig. 6D; Supplementary Fig. 7D). This observation shows the evolutionary origin of the main within-genome differences in gene expression (common for different cells), emphasizing the importance of evolutionary axis in transcriptome analyses.

### Other databases of PGCC transcriptomes

To check the generality of revealed effects, we studied the other available PGCC databases. The first additional database is the prostate cancer cell line (PPC1) with radiation-induced PGCC, treated or non-treated with pro-drug LCL521, which is an inhibitor of lysosomal enzyme acid ceramidase ASAH1 [29]. Both cases of PGCC show the similar movement to multicellularity (with suppression of stemness) along the evodevo axis, compared to initial cancer cells (Supplementary Figs. 9, 10).

The second database contains the two cell lines from ovarian cancers (Hey and SKOV3), where the PGCC were induced by treatment with tubulin-targeting drug paclitaxel [30]. Again, in both cell lines, there was the shift of PGCC to multicellularity (with the suppression of stemness), compared to initial cells (Supplementary Figs. 11, 12). For the SKOV3 line, the effect was somewhat smoothed, probably because these cells were harvested later (at 7 day after treatment vs. 3 day for Hey line) and may contain the progeny besides the PGCC. The progeny of PGCC from Hey cells show the backward movement to unicellularity and stemness, compared to PGCC (Supplementary Fig. 13), which is similar to the effect observed in the exemplar dataset (Fig. 8; Supplementary Fig. 7). The progeny of SKOV3 cells show an ambiguous picture (not shown), which supports the suggestion about a possible cell mixture in the PGCC sample.

The third additional database contains four permanent cell lines from ovarian and breast cancers (Hey, MCF7, OVCA432, SKOV3) and three primary cell lines (Org2414, Org2445, Org3008) from individual human high-grade serous ovarian cancers, cultivated in patient-derived xenografts [31]. In all cases, PGCC were induced by treatment with olaparib, which is an inhibitor of poly ADP-ribose polymerase (PARP). PGCC from Hey, MCF7, and Org2414 cells showed the shift to multicellularity, which is completely similar to previous cases (Supplementary Figs. 14–16). For Ovca432 and Org3008, the shift was also to multicellularity but the nucleus and plasma membrane genes showed an ambiguous picture (Supplementary Figs. 17, 18).

For Org2445 and SKOV3 cells, the 3-day cells (PGCC) were ambiguous (Supplementary Figs. 19, 20), whereas the 7-day cells (progeny) showed the movement to unicellularity and stemness (Supplementary Figs. 21, 22), which is similar to direction of movement of the late progeny in the exemplar case (Fig. 8; Suppl. Figure 7). Importantly, the PGCC's progeny of Org2445 and SKOV3 cells show the UC bias even compared with initial cells (Supplementary Figs. 21, 22), thus exceeding the movement of the late progeny in the exemplar case.

At last, the fourth additional database contains the three breast cancer cell lines (MDA, SUM159, Vari068), where PGCC were induced by treatment with cytoskeletal drug docetaxel [32]. PGCC of all three lines show a standard movement to multicellularity (with suppression of stemness), as in the most part of previous cases (Supplementary Figs. 23–25).

Thus, totally there were 15 datasets of independent experiments inducing PGCC in cancer cells of different origin by radiation or various chemical treatments, from which 13 experiments (87%) showed a clear movement to multicellularity (with suppression of stemness). In other three experiments, the results were ambiguous. From these three cases, one cell line (SKOV3), showing an ambiguous picture in one experiment, demonstrated a standard shift to multicellularity in other experiment (with other treatment). In the ambiguous experiment, the 3-day cells were ambiguous, while the later cells (PGCC's progeny) showed the shift to unicellularity. The same picture was observed for Org2445 cells. Therefore, we believe that the ambiguous cases can be explained by possible presence of cell mixtures with varying times of appearance of PGCC's progeny (especially, when different treatments and cell lines were used, which should add an additional variance in timing of PGCC and progeny origin). In the late progeny, the backward movement to unicellularity and stemness (i.e., to initial cancer cell state) took place (Fig. 8; Suppl. Figure 7). Possibly, at the beginning of trend reversal (from the shift to multicellularity in PGCC towards the opposite direction in the late progeny), the standard pattern of transcriptomic characteristics (e.g., Fig. 3B; Suppl. Figure 3B) became disturbing, with the temporal disbalance between the nucleus and plasma membrane genes and the evodevo signatures (Suppl. Figures 17, 18).

## Discussion

We found that in common polyploid cancer cells, which reach up to 56% in metastatic tumors [24], the genes of UC origin and stemness are upregulated, thus showing a shift to unicellularity along the evodevo axis, compared with diploid cancer cells. At that, the upregulated UC genes show a higher local and global protein interactome centrality than the upregulated stemness genes, which suggests that the UC attractor of cellular networks caused by the higher density of protein interactions in the ancient UC center [19] can be a driving force behind this movement along the evodevo axis. This is in agreement with the previous report that the UC signature is a stronger predictor of cancer than the stemness signature in cancer vs. normal cells while it is the other way round in prediction of stemness in normal stem vs. differentiated cells [22]. Surprisingly, the giant polyploid cancer cells (PGCC), which appear under severe stress in various cancers when the majority of cells die, showed the opposite picture. There occurs the suppression of UC and stemness genes (as well as nucleus-mapped genes) with the activation of MC genes (and cell membrane genes). This opposite movement along the evodevo axis is increased in the PGCC's early progeny but diminished in the late progeny, indicating its short-term nature.

Thus, PGCC present a unique reaction of cancer cells to severe stress. Usually, the progression of oncogenesis, stress, and polyploidization enhances the UC features of cancer cells because of a gradual loss of MC control and the activation of UC interactome attractor [19, 23], which is in agreement with the 'serial atavism' model [40]. Notably, genes that are damaged by somatic mutations driving oncogenesis (and thus presumably involved in control of cell cycle) mostly belong to early MC phylostrata [52, 53].

The enhanced adaptability of UC-like cancer cells is manifested in metastatic activity and resistance to treatment [5, 6, 8]. This is understandable because cancer cells, unleashing from MC control, begin to struggle for survival by all means (even at the expense of host), similarly to selfish UC organisms (e.g., UC parasites of MC hosts). However, it was emphasized that in this struggle, they can use not only the ancient UC mechanisms but also the MC heritage (reserved in the condensed chromatin) and even novel developments because selfish behavior determines only a general vector to survival but not necessarily its technical means [19, 22]. Therefore, if resistance acquired during the reversal to MC is beneficial for survival, it should retain even after the back-reversal to a UC-like state. The MC-biased state is used only for collective development of resistance due to cell cooperation (termed 'facilitation' in ecology), as was shown in works [54–56].

This usage of MC heritage is evident in metastatic cells that invade tissues other than a tissue of their origin. For instance, the metastatic cells invading the liver from colorectal cancer can adjust their metabolic pathways to suit the hepatic environment [57]. When the lung or breast cancer cells infiltrate the brain, most of them die. However, the remaining cells begin to express brain-specific protective factors, such as plasminogen activator inhibitory serpins, which allow them to survive [58]. These adaptations require the activation of tissue-specific pathways that were not specific to progenitors of metastatic cells. This is probably achieved via the epigenetic rewiring of cellular networks (partial transdifferentiation). Furthermore, adapting to their host as to external environment, cancer cells can use the MC heritage coercing the surrounding cells to assist in their survival. The examples include cancer-associated fibroblasts (CAF), tumor-associated macrophages (TAM), and angiogenesis [6, 8].

Even more spectacularly, the UC-like adaptability of cancer cells is manifested in the development of novel programs. This is needed because cancer cells can occur in altered (stressful) conditions, which were not previously encountered both in UC and MC life. Consequently, they must seek out novel solutions. Recent research revealed that cancer cells exhibit exploratory learning for adapting to unfamiliar conditions [59]. This learning is facilitated by epigenetic remodeling, representing a trial-and-error search for novel gene expression patterns. In metastasis and drug resistance, the adaptive transcriptional changes play a pivotal role [60]. Many such examples exist for UC fungi and cancer cells faced with not previously encountered drugs [61]. The resistance of cancer cells to chemicals, which were not previously encountered, was observed for a wide spectrum of drugs [5]. Importantly, we observed the activation of response to chemicals in the PGCC induced by radiation, which suggests the appearance of generalized stress resistance. In a similar vein, the activation of small heat shock proteins (CRYAA, CRYAB, CRYBB1), which are known as protectors against oxidative stress and apoptosis in normal and cancer cells [44, 45], can be considered as a hallmark of generalized stress resistance. These and certain other genes (CLDN6, CLDN19, SCN5A) can be used as biomarkers and therapeutic targets [49–51]. Probably, chemical stress follows radiation treatment because of cell damage.

An interesting parallel can be drawn from the yeast cells (that are real UC organisms). When faced with a significant challenge in a medium to which their biochemical networks were not adapted, they stop division but intensively metabolize [62]. Over time, they discover a network configuration suited to novel conditions and subsequently resume proliferation. This energetically demanding adaptation occurs via non-genetic mechanisms because the adaptation rate is orders of magnitude higher than expected based on known mutation rates [62]. Thus, it should be achieved by 'epigenetic learning'. This way of adaptation strikingly resembles the behavior of PGCC, which also stop division under severe stress until acquiring stress resistance, then resume proliferation. However, in contrast to the yeast, PGCC have at their disposal the MC heritage hidden in the condensed chromatin. The future studies of changes in chromatin accessibility near the corresponding MC genes could demonstrate the usage of MC heritage reserved in the conserved chromatin.

Here we showed that not only the atavistic shift to unicellularity but also the short-term return to multicellularity can take place in cancer cells under severe stress. Possibly, PGCC and their progeny may exploit the collective development of stress response associated with multicellularity. The collective cell response involves intensive intercellular communications for developing adaptation, which was called a 'hive mind' [54, 55, 63, 64]. It is also possible that for collective acquisition of stress resistance, the PGCC and their progeny undergo certain transdifferentiation because they activate the nervous-like pathways ('axon guidance') and develop cell extentions [28]. This phenotype resembles telocytes that also have long extentions (telopodes) and form the telopode-connected regulatory networks in the mesenchyme of various organs [65–69], which can be considered as a proto-nervous system. Besides cytokines, telocytes communicate via gap junctions (allowing a direct exchange of cytoplasmic substances between cells). We showed the gradual activation of gap junctions in the early PGCC progeny, with their suppression in the late progeny. The intercellular network communication might facilitate the collective development of stress resistance conferred to individual cells. Notably, under harsh conditions even prokaryotes can form drug-resistant MC-like structures (biofilms) [70].

Importantly, when resistance is acquired, the late progeny of PGCC return back to their selfish UC-like state. Albeit in the exemplar case of prostate cancer cell line (PPC1), where PGCC were obtained by radiation, the late progeny did not reach the initial level of unicellularity up to the latest time point that was studied (20 day), they may attain it later. Moreover, in the PGCC from Org2445 and SKOV3 cells obtained by olaparib treatment, the progeny of PGCC show even the higher UC bias than the initial cancer cells. This observation confirms that a general vector of cancer cell dynamics is towards unicellularity, in accordance with the 'serial atavism' model [40]. The serial atavism can be considered as a gradual downshifting along the complexity axis acquired during the MC evolution [71]. The relaxed MC control caused by this downshifting is seen in higher regenerative ability of lower animals [72–74]. However, in the case of severe stress, the general movement towards unicellularity might be interrupted by a short-term return to multicellularity facilitating collective development of stress resistance.

An intriguing evolutionary analogy can be drawn from the protists of the genus Dictyostelium. Under harsh conditions (starvation), these unicellular amoebas form giant cells with increased ploidy and MC structures with cell differentiation, which solve the problem of starvation by cannibalism of remaining single cells [75, 76]. When conditions become favorable, these giant cells and MC formations return back to a usual single-cell life [77]. Similarly, besides a transient shift to multicellularity, PGCC demonstrate cannibalism, engulfing neighboring small cells [26, 78], which suggests the awakening of ancient behavioral programs.

## Conclusions

While the common (ordinary) polyploid cancer cells comply with the 'serial atavism' model of oncogenesis, PGCC present a unique phenomenon of the short-term enhancement of multicellular features in cancers, probably associated with collective development of resistance to treatment. After the acquisition of resistance, PGCC's late progeny return back to a selfish UC-like state. Possibly, PGCC can be considered as a distorted recapitulation of the origin of MC organisms from individual cells in the evolution via polyploidization (caused by harsh conditions). The first principal component of transcriptome profiles, which is common for different cell types (initial cancer line, PGCC, early and late progeny) and contains the major part of gene expression variance, is also directed along the gene age axis. This finding reveals the evolutionary origin of the main within-genome differences in gene expression, emphasizing the importance of gene age axis in transcriptome analyses. Considering an entanglement of ontogenesis and phylogenesis along the evodevo axis, it should be pointed that phylogenesis is a primary cause of this axis formation, while ontogenesis only (partially) recapitulates it. The deep evolutionary basis of changes in gene expression across and within cell types might become a general framework for the interrelated problems of cell and cancer biology and regenerative medicine.

## Supplementary Information

Below is the link to the electronic supplementary material.

Supplementary Material 1

Supplementary Material 2

## Data Availability

Data from this study are provided in the main text and figures and Supplementary materials.

## References

[1] Siegel RL, Giaquinto AN, Jemal A. Cancer statistics, 2024. CA Cancer J Clin. 2024;74:12–49. 10.3322/caac.21820. 38230766 10.3322/caac.21820

[2] Cronin KA, Scott S, Firth AU, Sung H, Henley SJ, Sherman RL, et al. Annual report to the nation on the status of cancer, part 1: national cancer statistics. Cancer. 2022;128:4251–84. 10.1002/cncr.34479. 36301149 10.1002/cncr.34479 PMC10092838

[3] Sung H, Ferlay J, Siegel RL, Laversanne M, Soerjomataram I, Jemal A, et al. Global cancer statistics 2020: GLOBOCAN estimates of incidence and mortality worldwide for 36 cancers in 185 countries. CA Cancer J Clin. 2021;71:209–49. 10.3322/caac.21660. 33538338 10.3322/caac.21660

[4] Ugai T, Sasamoto N, Lee H-Y, Ando M, Song M, Tamimi RM, et al. Is early-onset cancer an emerging global epidemic? Current evidence and future implications. Nat Rev Clin Oncol. 2022;19:656–73. 10.1038/s41571-022-00672-8. 36068272 10.1038/s41571-022-00672-8 PMC9509459

[5] Vasan N, Baselga J, Hyman DM. A view on drug resistance in cancer. Nature. 2019;575:299–309. 10.1038/s41586-019-1730-1. 31723286 10.1038/s41586-019-1730-1 PMC8008476

[6] Eid RA, Alaa Edeen M, Shedid EM, Kamal ASS, Warda MM, Mamdouh F, et al. Targeting cancer stem cells as the key driver of carcinogenesis and therapeutic resistance. Int J Mol Sci. 2023;24:1786. 10.3390/ijms24021786. 36675306 10.3390/ijms24021786 PMC9861138

[7] Fatma H, Maurya SK, Siddique HR. Epigenetic modifications of c-MYC: role in cancer cell reprogramming, progression and chemoresistance. Semin Cancer Biol. 2022;83:166–76. 10.1016/j.semcancer.2020.11.008. 33220458 10.1016/j.semcancer.2020.11.008

[8] Toledo B, Picon-Ruiz M, Marchal JA, Perán M. Dual role of fibroblasts educated by tumour in cancer behavior and therapeutic perspectives. Int J Mol Sci. 2022;23:15576. 10.3390/ijms232415576. 36555218 10.3390/ijms232415576 PMC9778751

[9] Chen Y, Xue Y, Jin Y, Ji H. Lung stem cells in regeneration and tumorigenesis. J Genet Genomics. 2021;48:268–76. 10.1016/j.jgg.2020.12.004. 33896738 10.1016/j.jgg.2020.12.004

[10] Derks LLM, van Boxtel R. Stem cell mutations, associated cancer risk, and consequences for regenerative medicine. Cell Stem Cell. 2023;30:1421–33. 10.1016/j.stem.2023.09.008. 37832550 10.1016/j.stem.2023.09.008 PMC10624213

[11] Ebrahimi F, Pirouzmand F, Cosme Pecho RD, Alwan M, Yassen Mohamed M, Ali MS, et al. Application of mesenchymal stem cells in regenerative medicine: a new approach in modern medical science. Biotechnol Prog. 2023;39:e3374. 10.1002/btpr.3374. 37454344 10.1002/btpr.3374

[12] Wang Z. Assessing tumorigenicity in stem cell-derived therapeutic products: a critical step in safeguarding regenerative medicine. Bioengineering (Basel). 2023;10:857. 10.3390/bioengineering10070857. 37508884 10.3390/bioengineering10070857 PMC10376867

[13] Hanahan D. Hallmarks of cancer: new dimensions. Cancer Discov. 2022;12:31–46. 10.1158/2159-8290.CD-21-1059. 35022204 10.1158/2159-8290.CD-21-1059

[14] Hanahan D, Weinberg RA. The hallmarks of cancer. Cell. 2000;100:57–70. 10.1016/s0092-8674(00)81683-9. 10647931 10.1016/s0092-8674(00)81683-9

[15] Hanahan D, Weinberg RA. Hallmarks of cancer: the next generation. Cell. 2011;144:646–74. 10.1016/j.cell.2011.02.013. 21376230 10.1016/j.cell.2011.02.013

[16] Fouad YA, Aanei C. Revisiting the hallmarks of cancer. Am J Cancer Res. 2017;7:1016–36. 28560055 PMC5446472

[17] Senga SS, Grose RP. Hallmarks of cancer-the new testament. Open Biol. 2021;11:200358. 10.1098/rsob.200358. 33465324 10.1098/rsob.200358 PMC7881179

[18] Trigos AS, Pearson RB, Papenfuss AT, Goode DL. Altered interactions between unicellular and multicellular genes drive hallmarks of transformation in a diverse range of solid tumors. Proc Natl Acad Sci U S A. 2017;114:6406–11. 10.1073/pnas.1617743114. 28484005 10.1073/pnas.1617743114 PMC5474804

[19] Vinogradov AE, Anatskaya OV. Systemic alterations of cancer cells and their boost by polyploidization: unicellular attractor (UCA) model. IJMS. 2023;24:6196. 10.3390/ijms24076196. 37047167 10.3390/ijms24076196 PMC10094663

[20] Abzhanov A. Von Baer’s law for the ages: lost and found principles of developmental evolution. Trends Genet. 2013;29:712–22. 10.1016/j.tig.2013.09.004. 24120296 10.1016/j.tig.2013.09.004

[21] Vinogradov AE, Anatskaya OV. Cellular biogenetic law and its distortion by protein interactions: a possible unified framework for cancer biology and regenerative medicine. Int J Mol Sci. 2022;23:11486. 10.3390/ijms231911486. 36232785 10.3390/ijms231911486 PMC9570048

[22] Vinogradov AE, Anatskaya OV. “Cell dedifferentiation” versus “evolutionary reversal” theories of cancer: the direct contest of transcriptomic features. Int J Cancer. 2025;156:1802–13. 10.1002/ijc.35352. 39888036 10.1002/ijc.35352

[23] Quinton RJ, DiDomizio A, Vittoria MA, Kotýnková K, Ticas CJ, Patel S, et al. Whole-genome doubling confers unique genetic vulnerabilities on tumour cells. Nature. 2021;590:492–7. 10.1038/s41586-020-03133-3. 33505027 10.1038/s41586-020-03133-3 PMC7889737

[24] Priestley P, Baber J, Lolkema MP, Steeghs N, de Bruijn E, Shale C, et al. Pan-cancer whole-genome analyses of metastatic solid tumours. Nature. 2019;575:210–6. 10.1038/s41586-019-1689-y. 31645765 10.1038/s41586-019-1689-y PMC6872491

[25] Liu J. Giant cells: linking McClintock’s heredity to early embryogenesis and tumor origin throughout millennia of evolution on Earth. Semin Cancer Biol. 2022;81:176–92. 10.1016/j.semcancer.2021.06.007. 34116161 10.1016/j.semcancer.2021.06.007

[26] Liu P, Wang L, Yu H. Polyploid giant cancer cells: origin, possible pathways of formation, characteristics, and mechanisms of regulation. Front Cell Dev Biol. 2024;12:1410637. 10.3389/fcell.2024.1410637. 39055650 10.3389/fcell.2024.1410637 PMC11269155

[27] Song Y, Zhao Y, Deng Z, Zhao R, Huang Q. Stress-induced polyploid giant cancer cells: unique way of formation and non-negligible characteristics. Front Oncol. 2021;11:724781. 10.3389/fonc.2021.724781. 34527590 10.3389/fonc.2021.724781 PMC8435787

[28] White-Gilbertson S, Lu P, Saatci O, Sahin O, Delaney JR, Ogretmen B, et al. Transcriptome analysis of polyploid giant cancer cells and their progeny reveals a functional role for p21 in polyploidization and depolyploidization. J Biol Chem. 2024;300:107136. 10.1016/j.jbc.2024.107136. 38447798 10.1016/j.jbc.2024.107136 PMC10979113

[29] White-Gilbertson S, Lu P, Esobi I, Echesabal-Chen J, Mulholland PJ, Gooz M, et al. Polyploid giant cancer cells are dependent on cholesterol for progeny formation through amitotic division. Sci Rep. 2022;12:8971. 10.1038/s41598-022-12705-4. 35624221 10.1038/s41598-022-12705-4 PMC9142539

[30] Niu N, Yao J, Bast RC, Sood AK, Liu J. IL-6 promotes drug resistance through formation of polyploid giant cancer cells and stromal fibroblast reprogramming. Oncogenesis. 2021;10:65. 10.1038/s41389-021-00349-4. 34588424 10.1038/s41389-021-00349-4 PMC8481288

[31] Zhang X, Yao J, Li X, Niu N, Liu Y, Hajek RA, et al. Targeting polyploid giant cancer cells potentiates a therapeutic response and overcomes resistance to PARP inhibitors in ovarian cancer. Sci Adv. 2023;9:eadf7195. 10.1126/sciadv.adf7195. 37478190 10.1126/sciadv.adf7195 PMC10361597

[32] Zhou M, Ma Y, Chiang C-C, Rock EC, Butler SC, Anne R, et al. Single-cell morphological and transcriptome analysis unveil inhibitors of polyploid giant breast cancer cells in vitro. Commun Biol. 2023;6:1301. 10.1038/s42003-023-05674-5. 38129519 10.1038/s42003-023-05674-5 PMC10739852

[33] Vinogradov AE, Anatskaya OV. Growth of biological complexity from prokaryotes to hominids reflected in the human genome. Int J Mol Sci. 2021;22:11640. 10.3390/ijms222111640. 34769071 10.3390/ijms222111640 PMC8583824

[34] Domazet-Lošo T, Tautz D. A phylogenetically based transcriptome age index mirrors ontogenetic divergence patterns. Nature. 2010;468:815–8. 10.1038/nature09632. 21150997 10.1038/nature09632

[35] Barata T, Duarte I, Futschik ME. Integration of stemness gene signatures reveals core functional modules of stem cells and potential novel stemness genes. Genes. 2023;14:745. 10.3390/genes14030745. 36981016 10.3390/genes14030745 PMC10048104

[36] Yan Q, Fang X, Li C, Lan P, Guan X. Oncofetal proteins and cancer stem cells. Essays Biochem. 2022;66:423–33. 10.1042/EBC20220025. 35670043 10.1042/EBC20220025

[37] Szklarczyk D, Nastou K, Koutrouli M, Kirsch R, Mehryary F, Hachilif R, et al. The STRING database in 2025: protein networks with directionality of regulation. Nucleic Acids Res. 2025;53:D730–7. 10.1093/nar/gkae1113. 39558183 10.1093/nar/gkae1113 PMC11701646

[38] Franz M, Lopes CT, Fong D, Kucera M, Cheung M, Siper MC, et al. Cytoscape.js 2023 update: a graph theory library for visualization and analysis. Bioinformatics. 2023;39:btad031. 10.1093/bioinformatics/btad031. 36645249 10.1093/bioinformatics/btad031 PMC9889963

[39] Jayalath KP, Ng HKT. Analysis of means approach for random factor analysis. J Appl Stat. 2018;45:1426–46. 10.1080/02664763.2017.1375083.

[40] Lineweaver CH, Bussey KJ, Blackburn AC, Davies PCW. Cancer progression as a sequence of atavistic reversions. BioEssays. 2021;43:e2000305. 10.1002/bies.202000305. 33984158 10.1002/bies.202000305 PMC8860064

[41] Mirzayans R, Murray D. Amitotic cell division, malignancy, and resistance to anticancer agents: a tribute to Drs. Walen and Rajaraman. Cancers (Basel). 2024;16:3106. 10.3390/cancers16173106. 39272964 10.3390/cancers16173106 PMC11394378

[42] Tarkington J, Zhang H, Azevedo RBR, Zufall RA. Sex, amitosis, and evolvability in the ciliate *Tetrahymena thermophila*. Evolution. 2023;77:36–48. 10.1093/evolut/qpac031. 36622280 10.1093/evolut/qpac031

[43] Cowen L, Ideker T, Raphael BJ, Sharan R. Network propagation: a universal amplifier of genetic associations. Nat Rev Genet. 2017;18:551–62. 10.1038/nrg.2017.38. 28607512 10.1038/nrg.2017.38

[44] Cyran AM, Zhitkovich A. Heat shock proteins and HSF1 in cancer. Front Oncol. 2022;12:860320. 10.3389/fonc.2022.860320. 35311075 10.3389/fonc.2022.860320 PMC8924369

[45] Yin B, Tang S, Xu J, Sun J, Zhang X, Li Y, et al. CRYAB protects cardiomyocytes against heat stress by preventing caspase-mediated apoptosis and reducing F-actin aggregation. Cell Stress Chaperones. 2019;24:59–68. 10.1007/s12192-018-0941-y. 30246229 10.1007/s12192-018-0941-y PMC6363628

[46] Cheng L, Zou X, Wang J, Zhang J, Mo Z, Huang H. The role of CRYAB in tumor prognosis and immune infiltration: a pan-cancer analysis. Front Surg. 2022;9:1117307. 10.3389/fsurg.2022.1117307. 36713654 10.3389/fsurg.2022.1117307 PMC9880180

[47] Rashidieh B, Bain AL, Tria SM, Sharma S, Stewart CA, Simmons JL, et al. Alpha-B-crystallin overexpression is sufficient to promote tumorigenesis and metastasis in mice. Exp Hematol Oncol. 2023;12:4. 10.1186/s40164-022-00365-z. 36624493 10.1186/s40164-022-00365-z PMC9830749

[48] Xia H, Chen J, Zhang W, Xu Y, Nai Y, Wei X. CRYAB promotes colorectal cancer progression by inhibiting ferroptosis through blocking TRIM55-mediated β-catenin ubiquitination and degradation. Dig Dis Sci. 2024;69:3799–809. 10.1007/s10620-024-08584-6. 39126452 10.1007/s10620-024-08584-6 PMC11489300

[49] Ji W, Zhuang X, Jiang WG, Martin TA. Tight junctional protein family, claudins in cancer and cancer metastasis. Front Oncol. 2025;15:1596460. 10.3389/fonc.2025.1596460. 40687428 10.3389/fonc.2025.1596460 PMC12273447

[50] Liang Z, Tan W, Fang X, Zhang Z, Tan X, Zeng P. Multi-omics analysis revealed the diagnostic and therapeutic value of immunogenic cell death-derived SCN5A in hepatocellular carcinoma. Mol Biotechnol. 2025. 10.1007/s12033-025-01444-2. 40272736 10.1007/s12033-025-01444-2

[51] Sui Q, Peng J, Han K, Lin J, Zhang R, Ou Q, et al. Voltage-gated sodium channel Nav1.5 promotes tumor progression and enhances chemosensitivity to 5-fluorouracil in colorectal cancer. Cancer Lett. 2021;500:119–31. 10.1016/j.canlet.2020.12.017. 33338532 10.1016/j.canlet.2020.12.017

[52] Trigos AS, Pearson RB, Papenfuss AT, Goode DL. Somatic mutations in early metazoan genes disrupt regulatory links between unicellular and multicellular genes in cancer. Elife. 2019;8:e40947. 10.7554/eLife.40947. 30803482 10.7554/eLife.40947 PMC6402835

[53] Trigos AS, Bongiovanni F, Zhang Y, Zethoven M, Tothill R, Pearson R, et al. Disruption of metazoan gene regulatory networks in cancer alters the balance of co-expression between genes of unicellular and multicellular origins. Genome Biol. 2024;25:110. 10.1186/s13059-024-03247-1. 38685127 10.1186/s13059-024-03247-1 PMC11057133

[54] Bhattacharya S, Mohanty A, Achuthan S, Kotnala S, Jolly MK, Kulkarni P, et al. Group behavior and emergence of cancer drug resistance. Trends Cancer. 2021;7:323–34. 10.1016/j.trecan.2021.01.009. 33622644 10.1016/j.trecan.2021.01.009 PMC8500356

[55] Emond R, Griffiths JI, Grolmusz VK, Nath A, Chen J, Medina EF, et al. Cell facilitation promotes growth and survival under drug pressure in breast cancer. Nat Commun. 2023;14:3851. 10.1038/s41467-023-39242-6. 37386030 10.1038/s41467-023-39242-6 PMC10310817

[56] Venkataramani V, Schneider M, Giordano FA, Kuner T, Wick W, Herrlinger U, et al. Disconnecting multicellular networks in brain tumours. Nat Rev Cancer. 2022;22:481–91. 10.1038/s41568-022-00475-0. 35488036 10.1038/s41568-022-00475-0

[57] Loo JM, Scherl A, Nguyen A, Man FY, Weinberg E, Zeng Z, et al. Extracellular metabolic energetics can promote cancer progression. Cell. 2015;160:393–406. 10.1016/j.cell.2014.12.018. 25601461 10.1016/j.cell.2014.12.018 PMC4312495

[58] Valiente M, Obenauf AC, Jin X, Chen Q, Zhang XH-F, Lee DJ, et al. Serpins promote cancer cell survival and vascular co-option in brain metastasis. Cell. 2014;156:1002–16. 10.1016/j.cell.2014.01.040. 24581498 10.1016/j.cell.2014.01.040 PMC3988473

[59] Shomar A, Barak O, Brenner N. Cancer progression as a learning process. iScience. 2022;25:103924. 10.1016/j.isci.2022.103924. 35265809 10.1016/j.isci.2022.103924 PMC8898914

[60] Ganesh K, Massagué J. Targeting metastatic cancer. Nat Med. 2021;27:34–44. 10.1038/s41591-020-01195-4. 33442008 10.1038/s41591-020-01195-4 PMC7895475

[61] El Meouche I, Jain P, Jolly MK, Capp J-P. Drug tolerance and persistence in bacteria, fungi and cancer cells: role of non-genetic heterogeneity. Transl Oncol. 2024;49:102069. 10.1016/j.tranon.2024.102069. 39121829 10.1016/j.tranon.2024.102069 PMC11364053

[62] Woronoff G, Nghe P, Baudry J, Boitard L, Braun E, Griffiths AD, et al. Metabolic cost of rapid adaptation of single yeast cells. Proc Natl Acad Sci USA. 2020;117:10660–6. 10.1073/pnas.1913767117. 32371488 10.1073/pnas.1913767117 PMC7245094

[63] Davis JB, Krishna SS, Abi Jomaa R, Duong CT, Espina V, Liotta LA, et al. A new model isolates glioblastoma clonal interactions and reveals unexpected modes for regulating motility, proliferation, and drug resistance. Sci Rep. 2019;9:17380. 10.1038/s41598-019-53850-7. 31758030 10.1038/s41598-019-53850-7 PMC6874607

[64] Williams JB, Li S, Higgs EF, Cabanov A, Wang X, Huang H, et al. Tumor heterogeneity and clonal cooperation influence the immune selection of IFN-γ-signaling mutant cancer cells. Nat Commun. 2020;11:602. 10.1038/s41467-020-14290-4. 32001684 10.1038/s41467-020-14290-4 PMC6992737

[65] Kaestner KH. The intestinal stem cell niche: a central role for Foxl1-expressing subepithelial telocytes. Cell Mol Gastroenterol Hepatol. 2019;8:111–7. 10.1016/j.jcmgh.2019.04.001. 30965141 10.1016/j.jcmgh.2019.04.001 PMC6538877

[66] Li F, Tang X, Cao H, Wang W, Geng C, Sun Z, et al. Vascular endothelial growth factor facilitates the effects of telocytes on tumor cell proliferation and migration. Front Cell Dev Biol. 2024;12:1474682. 10.3389/fcell.2024.1474682. 39605983 10.3389/fcell.2024.1474682 PMC11599237

[67] Purelku M, Sahin H, Erkanli Senturk G, Tanriverdi G. Distribution and morphologic characterization of telocytes in rat ovary and uterus: insights from ultrastructural and immunohistochemical analysis. Histochem Cell Biol. 2024;162:373–84. 10.1007/s00418-024-02313-w. 39078438 10.1007/s00418-024-02313-w PMC11393091

[68] Sanches BDA, Rocha LC, Neto JP, Beguelini MR, Ciena AP, Carvalho HF. Telocytes of the male reproductive system: dynamic tissue organizers. Front Cell Dev Biol. 2024;12:1444156. 10.3389/fcell.2024.1444156. 39469114 10.3389/fcell.2024.1444156 PMC11513265

[69] Zhang J, Xu Y. Tumor-associated telocytes. Chin Med J (Engl). 2024;137:490–2. 10.1097/CM9.0000000000003016. 38238151 10.1097/CM9.0000000000003016 PMC10876250

[70] Panda SK, da Silva LCN. Editorial: understanding biofilms: recent trends and developments. Front Cell Infect Microbiol. 2025;15:1566230. 10.3389/fcimb.2025.1566230. 40007611 10.3389/fcimb.2025.1566230 PMC11850994

[71] Vinogradov AE, Anatskaya OV. Gradistics: an underappreciated dimension in evolutionary space. Biosystems. 2023;224:104844. 10.1016/j.biosystems.2023.104844. 36736879 10.1016/j.biosystems.2023.104844

[72] Corradetti B, Dogra P, Pisano S, Wang Z, Ferrari M, Chen S-H, et al. Amphibian regeneration and mammalian cancer: similarities and contrasts from an evolutionary biology perspective—comparing the regenerative potential of mammalian embryos and urodeles to develop effective strategies against human cancer. BioEssays. 2021;43:e2000339. 10.1002/bies.202000339. 33751590 10.1002/bies.202000339

[73] Khyeam S, Lee S, Huang GN. Genetic, epigenetic, and post-transcriptional basis of divergent tissue regenerative capacities among vertebrates. Adv Genet. 2021;2:e10042. 10.1002/ggn2.10042. 34423307 10.1002/ggn2.10042 PMC8372189

[74] Nguyen PD, de Bakker DEM, Bakkers J. Cardiac regenerative capacity: an evolutionary afterthought? Cell Mol Life Sci. 2021;78:5107–22. 10.1007/s00018-021-03831-9. 33950316 10.1007/s00018-021-03831-9 PMC8254703

[75] Amagai A. Involvement of a novel gene, zyg1, in zygote formation of *Dictyostelium mucoroides*. J Muscle Res Cell Motil. 2002;23:867–74. 10.1023/a:1024448316675. 12952084 10.1023/a:1024448316675

[76] Bloomfield G. Sex and macrocyst formation in Dictyostelium. Int J Dev Biol. 2019;63:439–46. 10.1387/ijdb.190183gb. 31840782 10.1387/ijdb.190183gb

[77] Shibasaki S, Shimada M. Cyclic dominance emerges from the evolution of two inter-linked cooperative behaviours in the social amoeba. Proc Biol Sci. 2018;285:20180905. 10.1098/rspb.2018.0905. 29925622 10.1098/rspb.2018.0905 PMC6030527

[78] Casotti MC, Meira DD, Zetum ASS, de Araújo BC, da Silva DRC, de Santos E, et al. Computational biology helps understand how polyploid giant cancer cells drive tumor success. Genes (Basel). 2023;14:801. 10.3390/genes14040801. 37107559 10.3390/genes14040801 PMC10137723

