# Supplementary material for: Polyploid giant cancer cells (PGCC): short-term return to multicellularity
Source: Biol Res. 2025 Nov 24;58:71. doi: 10.1186/s40659-025-00650-1 (PMC12642267; doi:10.1186/s40659-025-00650-1)
Supplement: Supplementary file 1 — Supplementary Material 1 [file 40659_2025_650_MOESM1_ESM.pdf]

Supplementary Figures to

A.E. Vinogradov and O.V. Anatskaya

Polyploid giant cancer cells (PGCC): short-term return to multicellularity

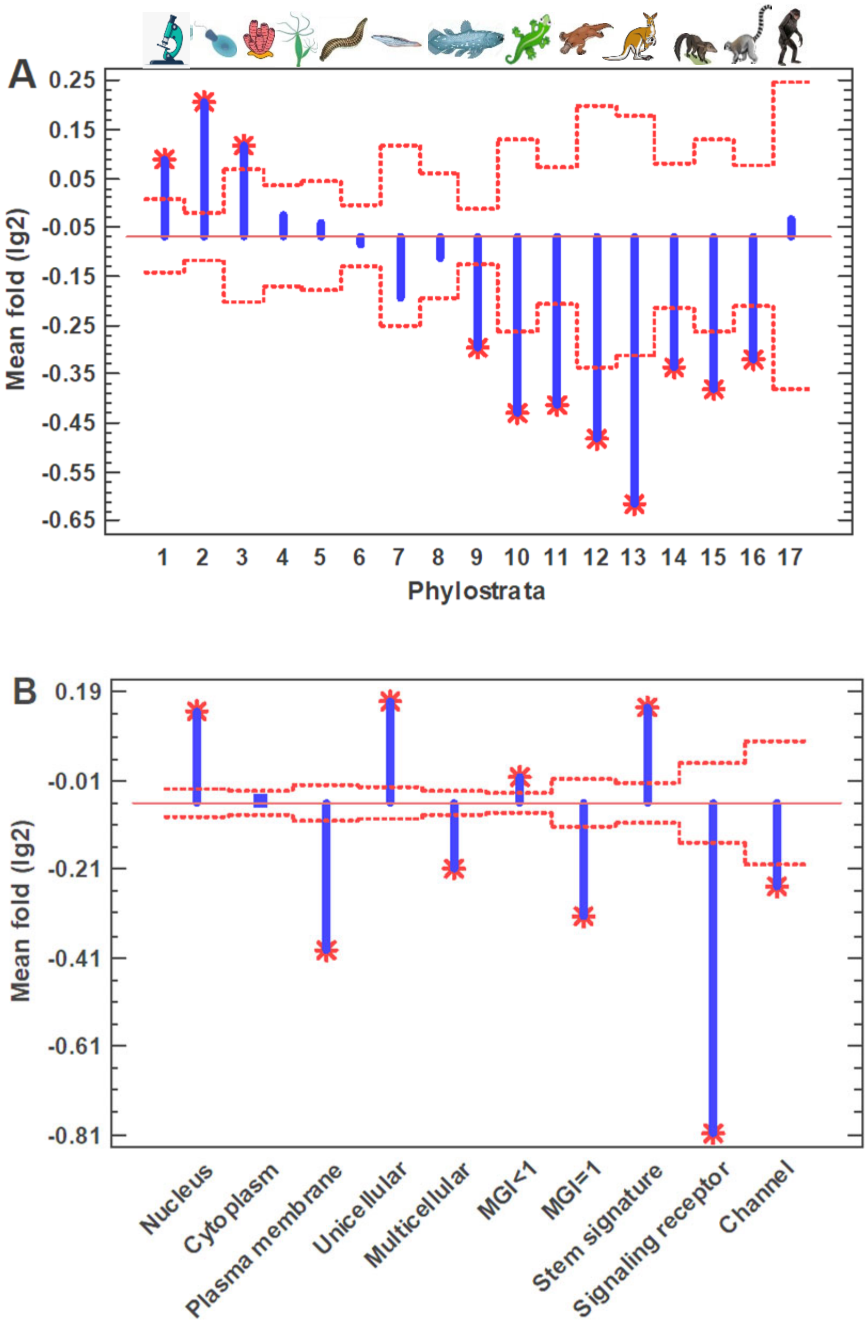

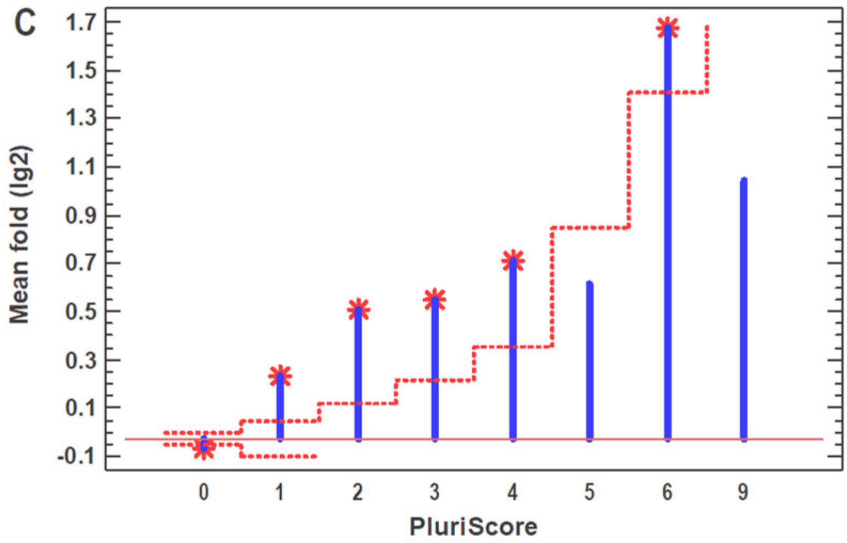

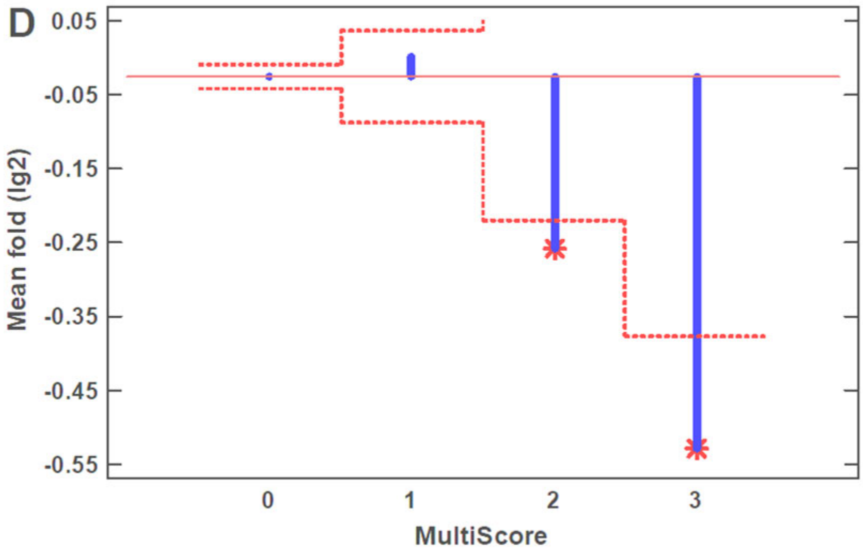

Suppl. Fig. 1. Characteristics of common polyploid cancer cells (compared to diploid cancer cells), **with cell cycle genes excluded**. **A** -- Evolutionary profile of gene expression fold. Phylostrata: 1—cellular organisms (Prokaryota); 2—Eukaryota; 3—Opisthokonta; 4—Metazoa; 5—Eumetazoa; 6—Bilateria; 7—Chordata; 8—Vertebrata; 9—Euteleostomi; 10—Tetrapoda; 11—Amniota; 12—Mammalia; 13—Theria; 14—Eutheria; 15—Boreoeutheria; 16—Primates; 17—Hominidae. (First three phylostrata are unicellular. The pictures at the top show recent organisms corresponding to phyletic branching used for human gene dating.) **B** – Gene expression folds for different signatures (MGI, multicellularity gene index). **C** -- Gene expression folds for pluripotent genes (PluriScore, the number of pluripotent cell databases, where a gene is present). **D** -- Gene expression folds for multipotent genes (MultiScore, the number of multipotent cell databases, where a gene is present). Red dotted lines show confidence intervals (p=0.05), red stars – significant differences.

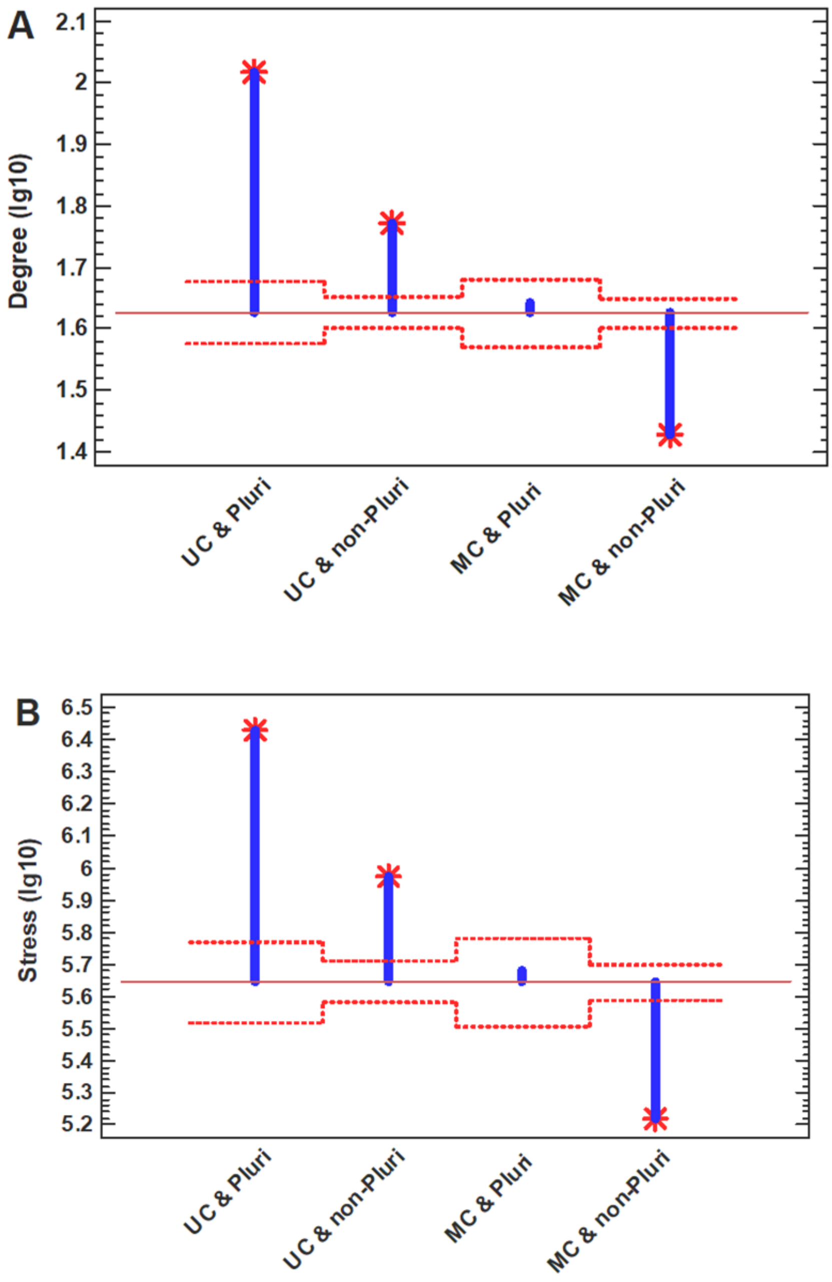
A
Degree (lg10)
2.1
2
1.9
1.8
1.7
1.6
1.5
1.4
UC & Pluri
UC & non-Pluri
MC & Pluri
MC & non-Pluri
B
Stress (lg10)
6.5
6.4
6.3
6.2
6.1
6
5.9
5.8
5.7
5.6
5.5
5.4
5.3
5.2
UC & Pluri
UC & non-Pluri
MC & Pluri
MC & non-Pluri

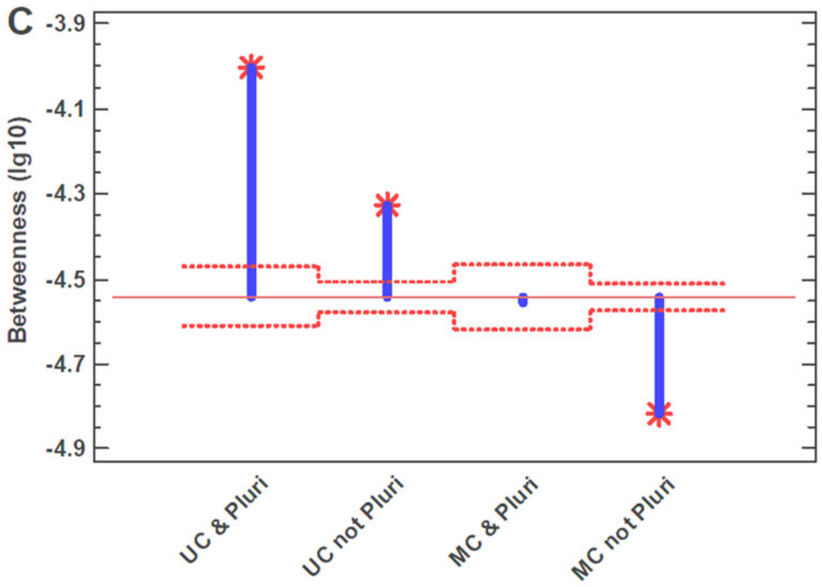

Suppl. Fig. 2. Protein interactome centrality measures for the upregulated genes in common polyploid cancer cells (compared to diploid cancer cells), **with cell cycle genes excluded**. **A** – Degree, the number of direct (one-step) interactions of a given protein (local centrality measure). **B** – Stress, the total number of shortest paths between all pairs of other proteins passing through a given protein (global centrality measure showing load or traffic on a network node). **C** – Betweenness, similar to stress but paths are weighted by inverse of total paths (global centrality measure showing control or brokerage role of a node in the network). Red dotted lines show confidence intervals (p=0.05), red stars – significant differences.

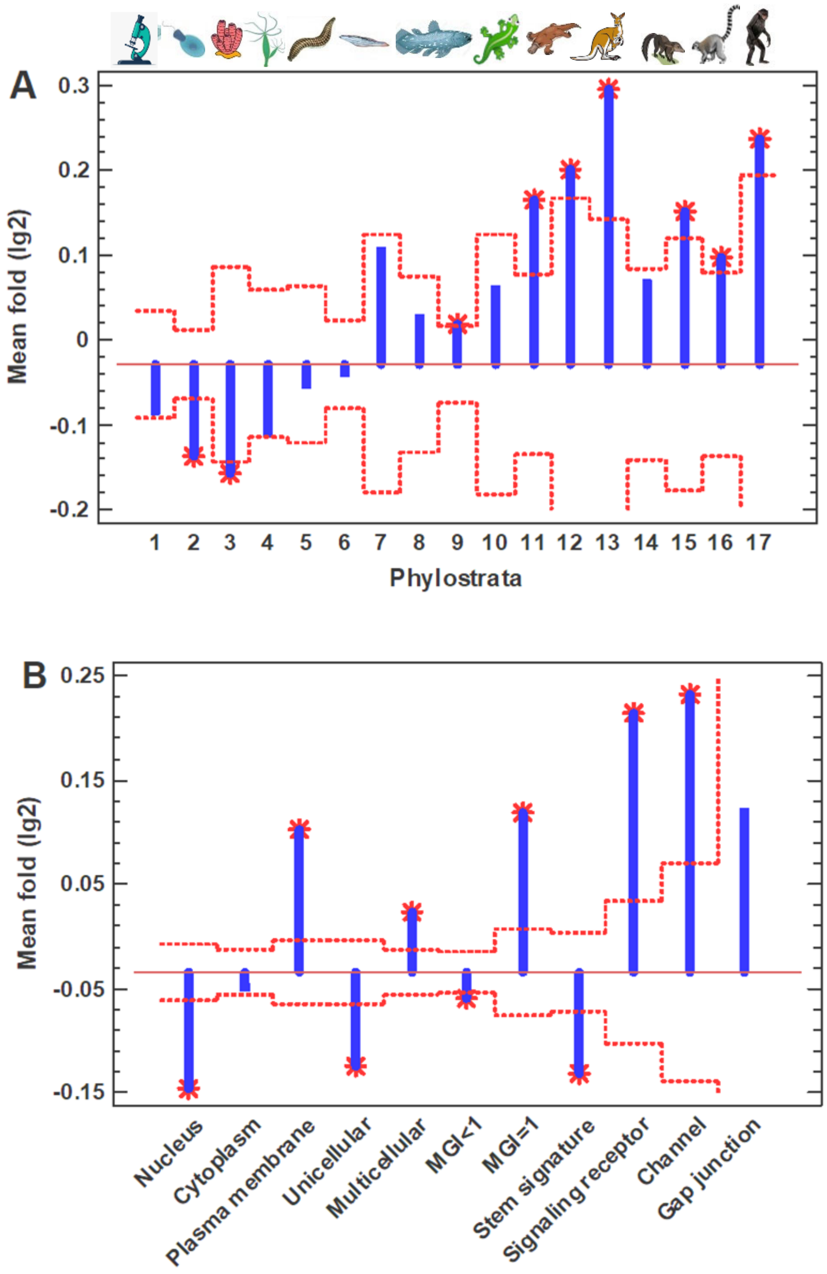
A
0.3
0.2
0.1
0
-0.1
-0.2
Mean fold (lg2)
1 2 3 4 5 6 7 8 9 10 11 12 13 14 15 16 17
Phylostrata
B
0.25
0.15
0.05
-0.05
-0.15
Mean fold (lg2)
Nucleus
Cytoplasm
Plasma membrane
Unicellular
Multicellular
MGI<1
MGI=1
Stem signature
Signaling receptor
Channel
Gap junction

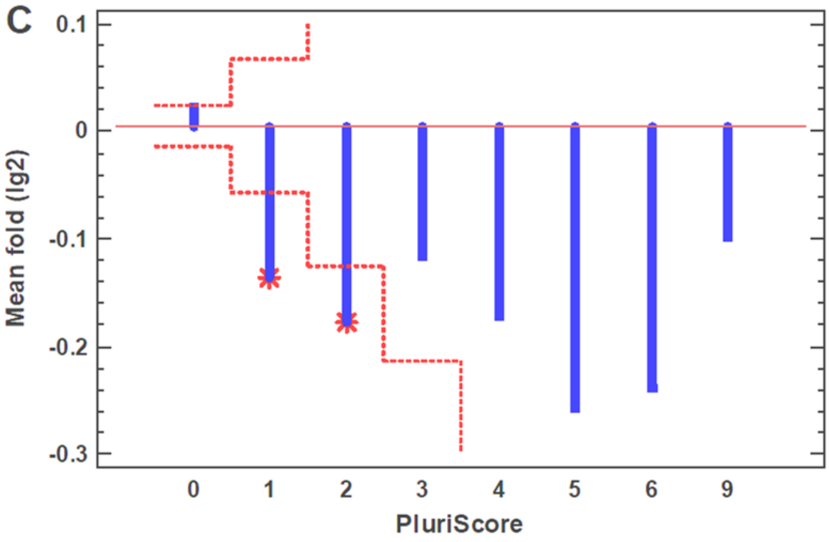

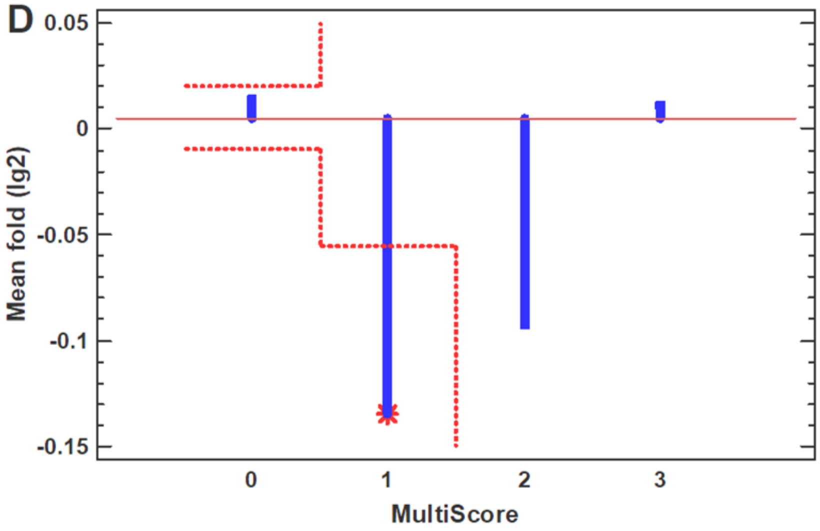

Suppl. Fig. 3. Characteristics of PGCC, compared to initial cancer cells (from GSE196453), **with cell cycle genes excluded**. **A** -- Evolutionary profile of gene expression fold. Phylostrata: 1—cellular organisms (Prokaryota); 2—Eukaryota; 3—Opisthokonta; 4—Metazoa; 5—Eumetazoa; 6—Bilateria; 7—Chordata; 8—Vertebrata; 9—Euteleostomi; 10—Tetrapoda; 11—Amniota; 12—Mammalia; 13—Theria; 14—Eutheria; 15—Boreoeutheria; 16—Primates; 17—Hominidae. (First three phylostrata are unicellular. The pictures at the top show recent organisms corresponding to phyletic branching used for human gene dating.) **B** – Gene expression folds for different signatures (MGI, multicellularity gene index). **C** -- Gene expression folds for pluripotent genes (PluriScore, the number of pluripotent cell databases, where a gene is present). **D** -- Gene expression folds for multipotent genes (MultiScore, the number of multipotent cell databases, where a gene is present). Red dotted lines show confidence intervals (p=0.05), red stars – significant differences.

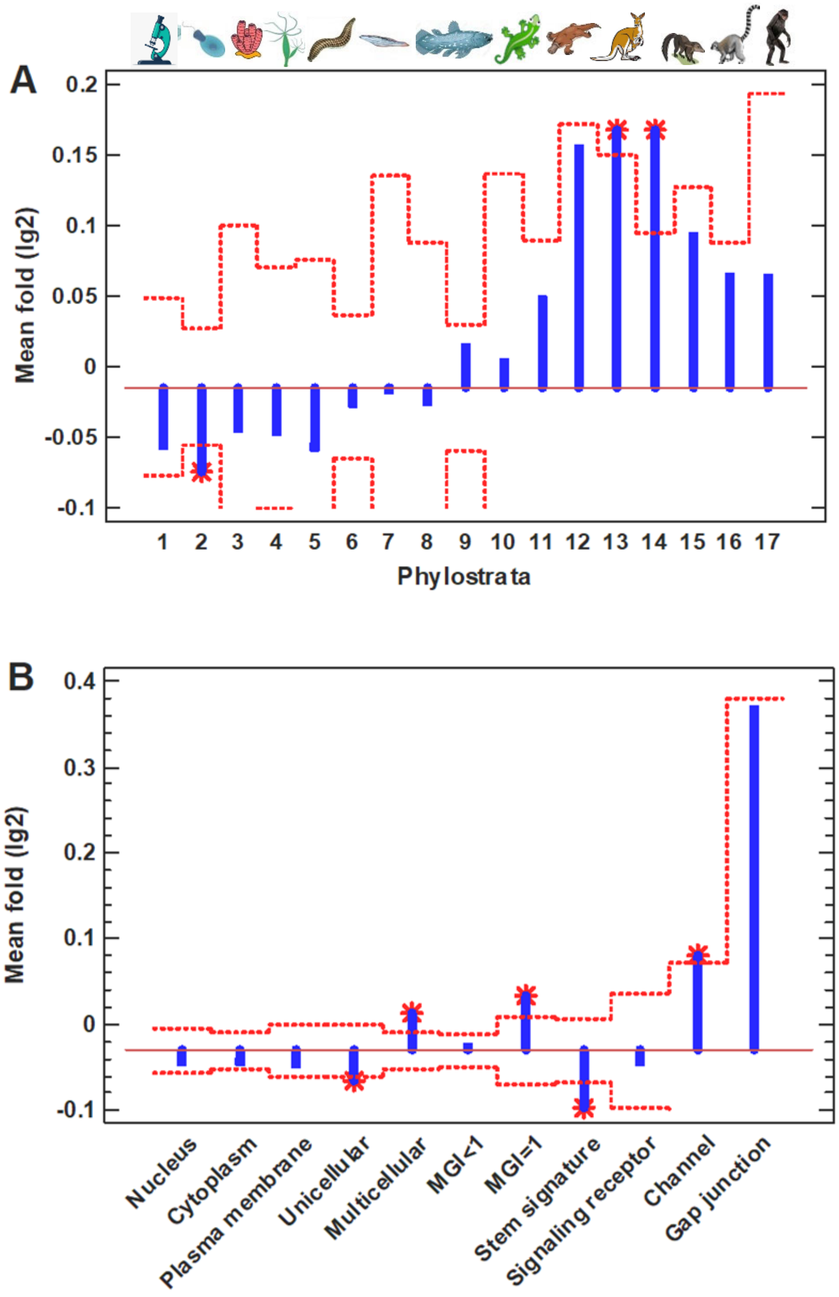
A
Mean fold (lg2)
0.2
0.15
0.1
0.05
0
-0.05
-0.1
1 2 3 4 5 6 7 8 9 10 11 12 13 14 15 16 17
Phylostrata
B
Mean fold (lg2)
0.4
0.3
0.2
0.1
0
-0.1
Nucleus
Cytoplasm
Plasma membrane
Unicellular
Multicellular
MGI<1
MGI=1
Stem signature
Signaling receptor
Channel
Gap junction

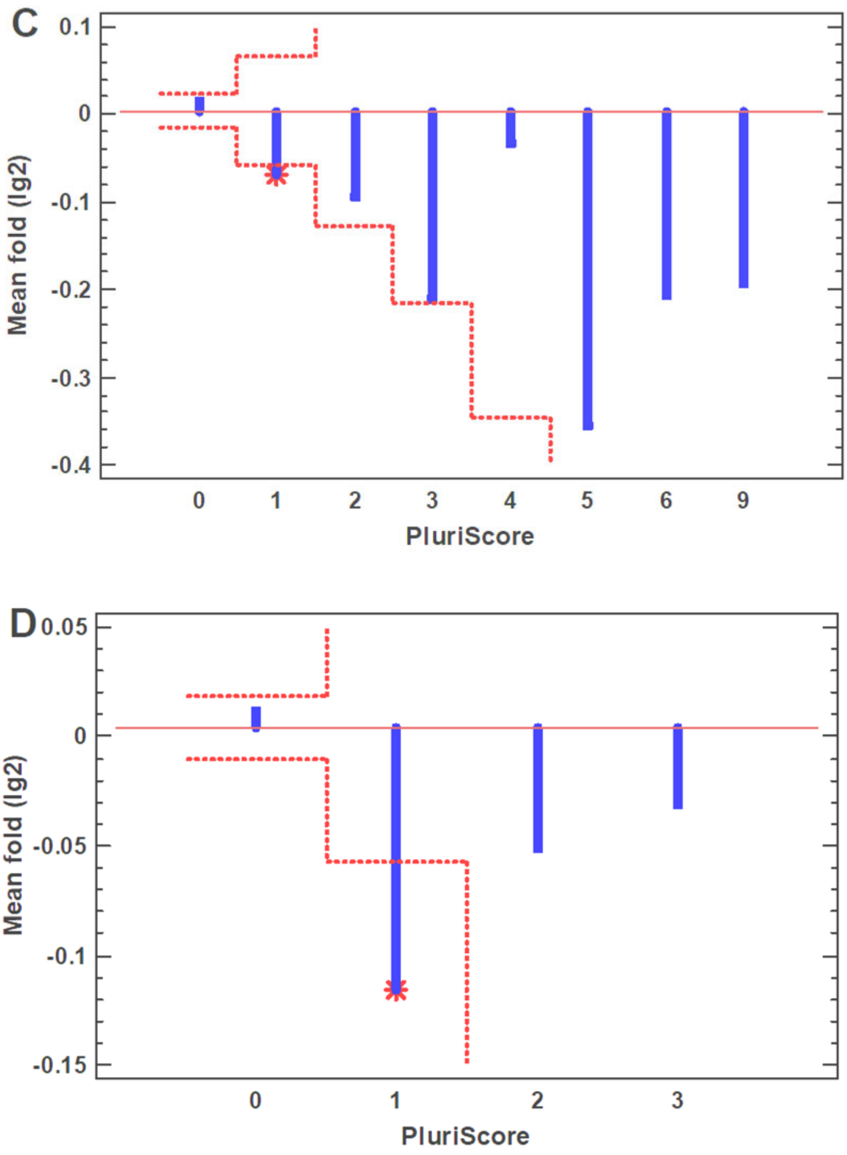

Suppl. Fig. 4. Characteristics of PGCC's early progeny, compared to PGCC (from GSE196453), with cell cycle genes excluded. **A** -- Evolutionary profile of gene expression fold. Phylostrata: 1—cellular organisms (Prokaryota); 2—Eukaryota; 3—Opisthokonta; 4—Metazoa; 5—Eumetazoa; 6—Bilateria; 7—Chordata; 8—Vertebrata; 9—Euteleostomi; 10—Tetrapoda; 11—Amniota; 12—Mammalia; 13—Theria; 14—Eutheria; 15—Boreoeutheria; 16—Primates; 17—Hominidae. (First three phylostrata are unicellular. The pictures at the top show recent organisms corresponding to phyletic branching used for human gene dating.) **B** – Gene expression folds for different signatures (MGI, multicellularity gene index). **C** -- Gene expression folds for pluripotent genes (PluriScore, the number of pluripotent cell databases, where a gene is present). **D** -- Gene expression folds for multipotent genes (MultiScore, the number of multipotent cell databases, where a gene is present). Red dotted lines show confidence intervals (*p*=0.05), red stars – significant differences.

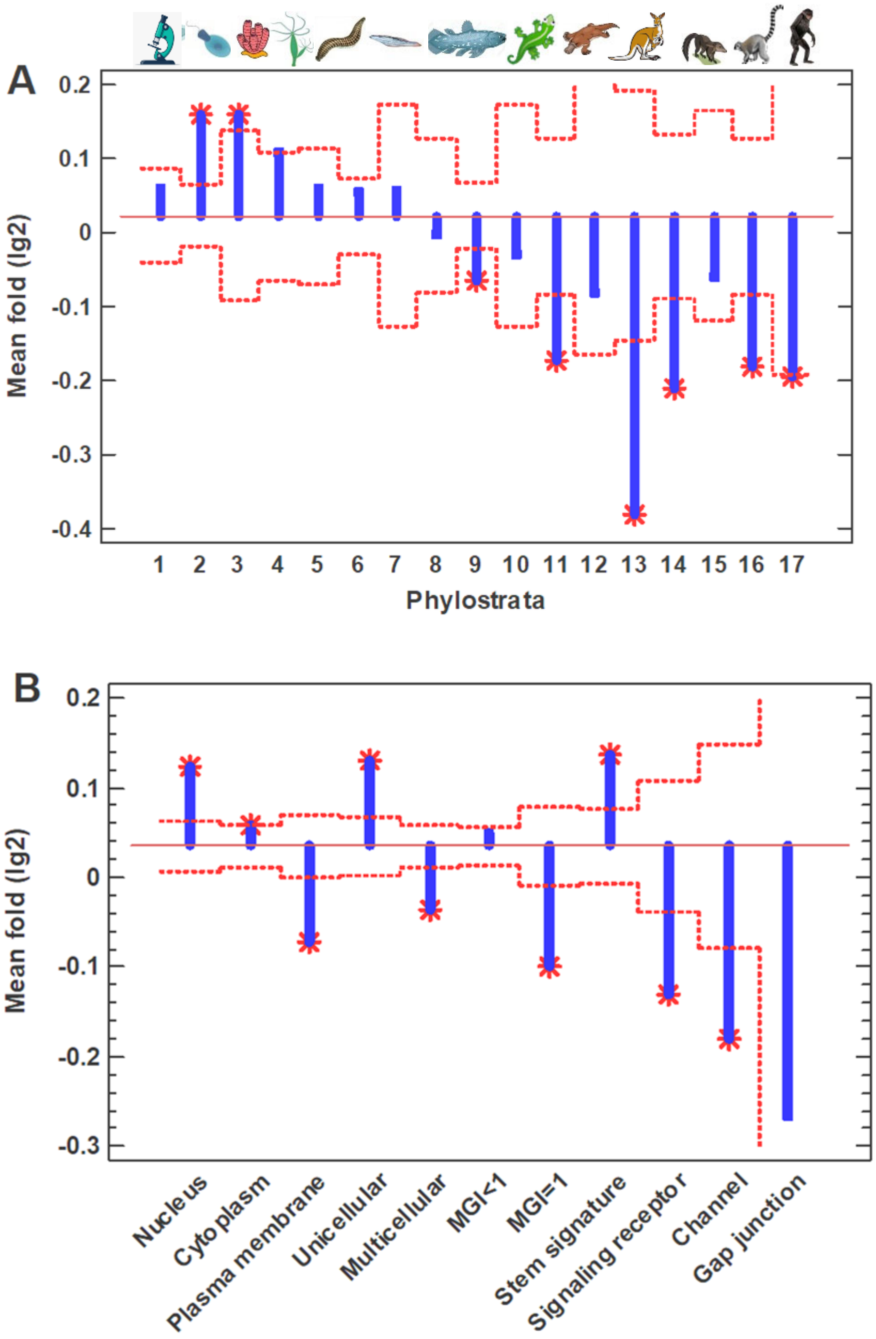
A
0.2
0.1
0
-0.1
-0.2
-0.3
-0.4
Mean fold (lg2)
1 2 3 4 5 6 7 8 9 10 11 12 13 14 15 16 17
Phylostrata
B
0.2
0.1
0
-0.1
-0.2
-0.3
Mean fold (lg2)
Nucleus
Cyto plasm
Plasma membrane
Unicellular
Multicellular
MGI<1
MGI=1
Stem signature
Signaling recepto r
Channel
Gap junction

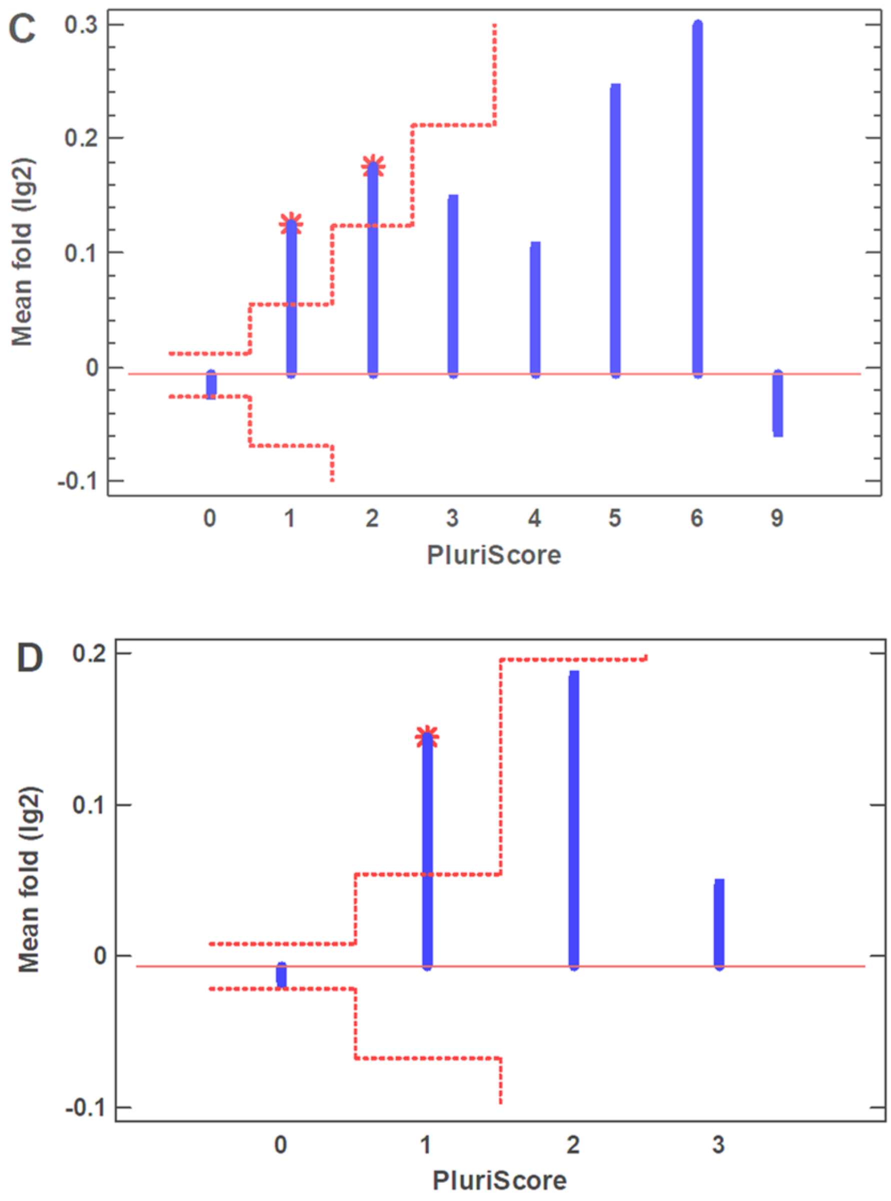

Suppl. Fig. 5. Characteristics of PGCC's late progeny, compared to the early progeny (from GSE196453), with cell cycle genes excluded. **A** -- Evolutionary profile of gene expression fold. Phylostrata: 1—cellular organisms (Prokaryota); 2—Eukaryota; 3—Opisthokonta; 4—Metazoa; 5—Eumetazoa; 6—Bilateria; 7—Chordata; 8—Vertebrata; 9—Euteleostomi; 10—Tetrapoda; 11—Amniota; 12—Mammalia; 13—Theria; 14—Eutheria; 15—Boreoeutheria; 16—Primates; 17—Hominidae. (First three phylostrata are unicellular. The pictures at the top show recent organisms corresponding to phyletic branching used for human gene dating.) **B** – Gene expression folds for different signatures (MGI, multicellularity gene index). **C** -- Gene expression folds for pluripotent genes (PluriScore, the number of pluripotent cell databases, where a gene is present). **D** -- Gene expression folds for multipotent genes (MultiScore, the number of multipotent cell databases, where a gene is present). Red dotted lines show confidence intervals (*p*=0.05), red stars – significant differences.

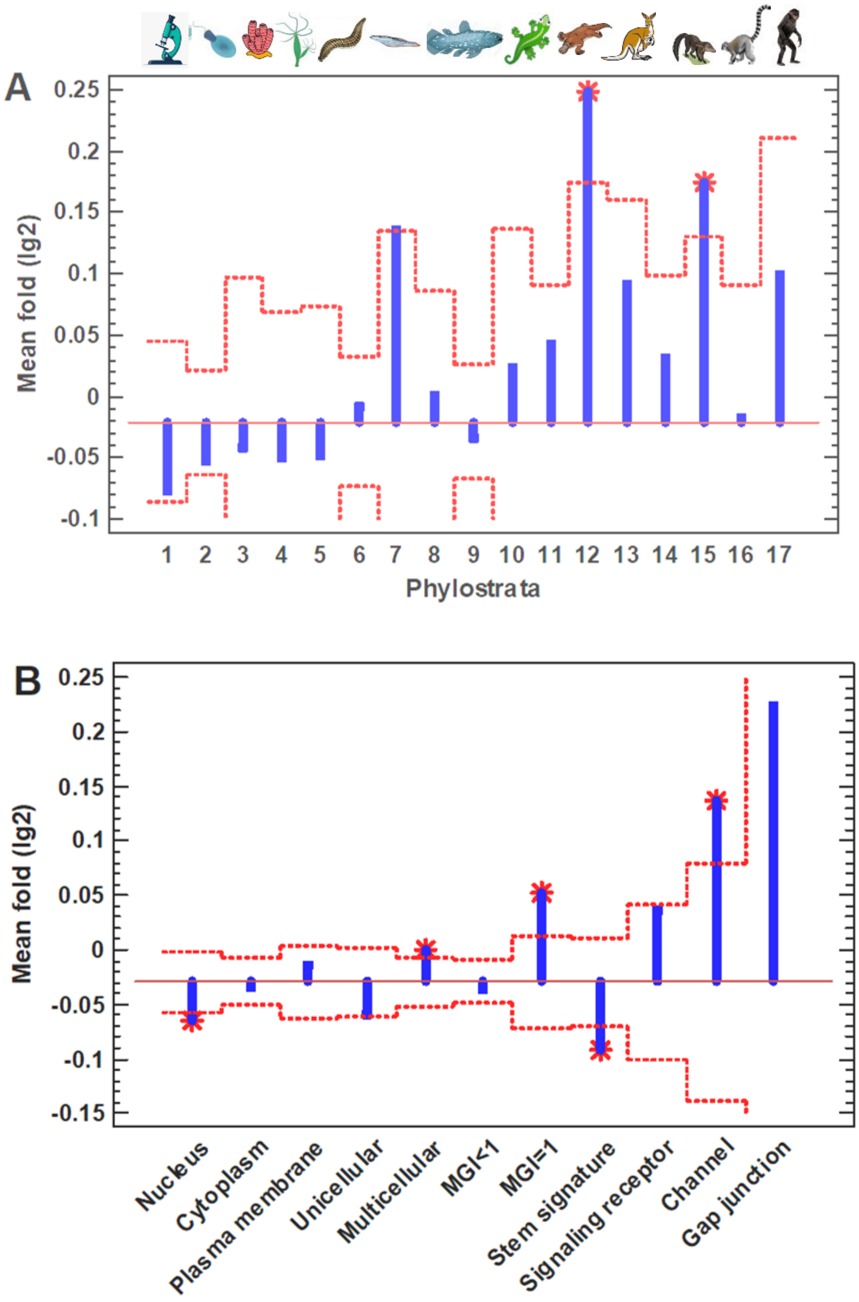
A
0.25
0.2
0.15
0.1
0.05
0
-0.05
-0.1
Mean fold (lg2)
1 2 3 4 5 6 7 8 9 10 11 12 13 14 15 16 17
Phylostrata
B
0.25
0.2
0.15
0.1
0.05
0
-0.05
-0.1
-0.15
Mean fold (lg2)
Nucleus
Cytoplasm
Plasma membrane
Unicellular
Multicellular
MGI<1
MGI=1
Stem signature
Signaling receptor
Channel
Gap junction

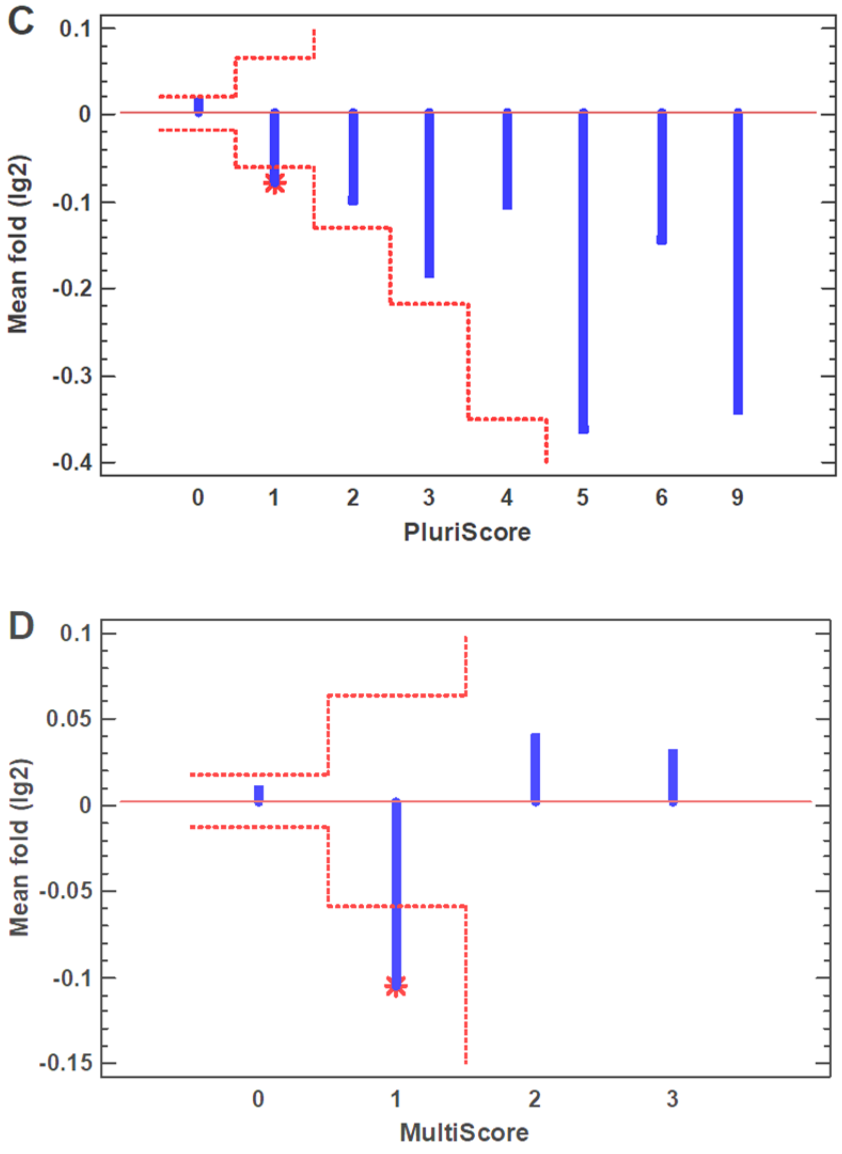

Suppl. Fig. 6. Characteristics of PGCC's late progeny, compared to initial cancer cells (from GSE196453), with cell cycle genes excluded. **A** -- Evolutionary profile of gene expression fold. Phylostrata: 1—cellular organisms (Prokaryota); 2—Eukaryota; 3—Opisthokonta; 4—Metazoa; 5—Eumetazoa; 6—Bilateria; 7—Chordata; 8—Vertebrata; 9—Euteleostomi; 10—Tetrapoda; 11—Amniota; 12—Mammalia; 13—Theria; 14—Eutheria; 15—Boreoeutheria; 16—Primates; 17—Hominidae. (First three phylostrata are unicellular. The pictures at the top show recent organisms corresponding to phyletic branching used for human gene dating.) **B** – Gene expression folds for different signatures (MGI, multicellularity gene index). **C** -- Gene expression folds for pluripotent genes (PluriScore, the number of pluripotent cell databases, where a gene is present). **D** -- Gene expression folds for multipotent genes (MultiScore, the number of multipotent cell databases, where a gene is present). Red dotted lines show confidence intervals (p=0.05), red stars – significant differences.

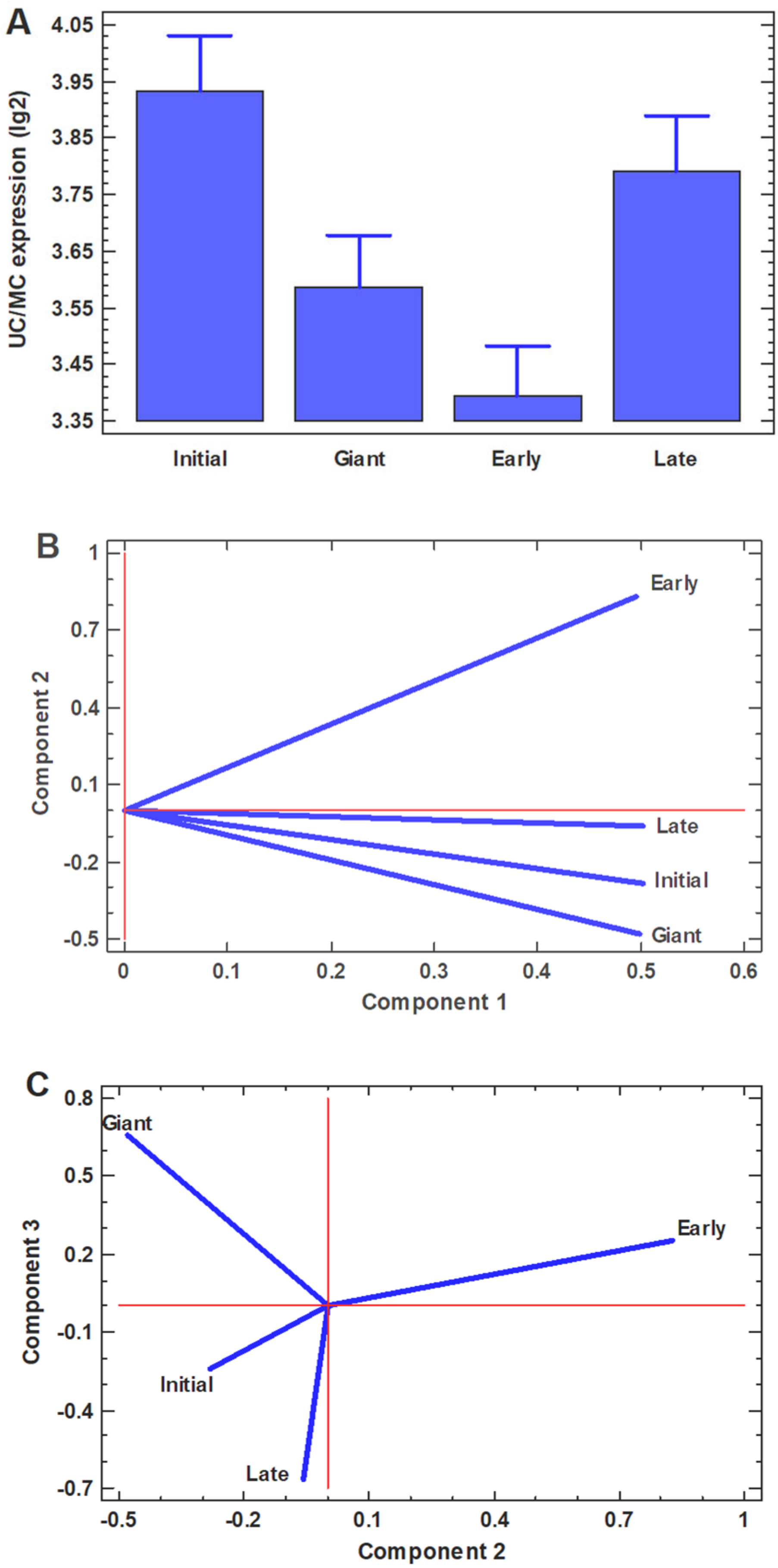
A
UC/MC expression (lg2)
4.05
3.95
3.85
3.75
3.65
3.55
3.45
3.35
Initial
Giant
Early
Late
B
Component 2
1
0.7
0.4
0.1
-0.2
-0.5
0
0.1
0.2
0.3
0.4
0.5
0.6
Component 1
Early
Late
Initial
Giant
C
Component 3
0.8
0.5
0.2
-0.1
-0.4
-0.7
-0.5
-0.2
0.1
0.4
0.7
1
Component 2
Giant
Early
Initial
Late

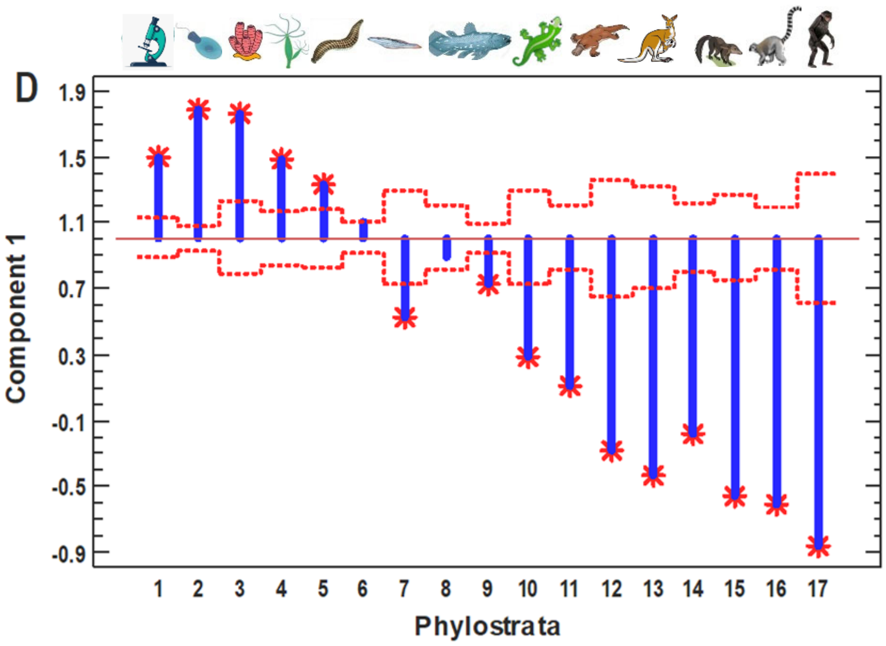

Suppl. Fig. 7. General picture for all four cell types (initial cancer cells, PGCC, their early and later progeny), **with cell cycle genes excluded** (from GSE196453). **A** -- UC/MC gene expression ratio; **B, C, D** – principal component analysis (PCA) of the whole transcriptomes.

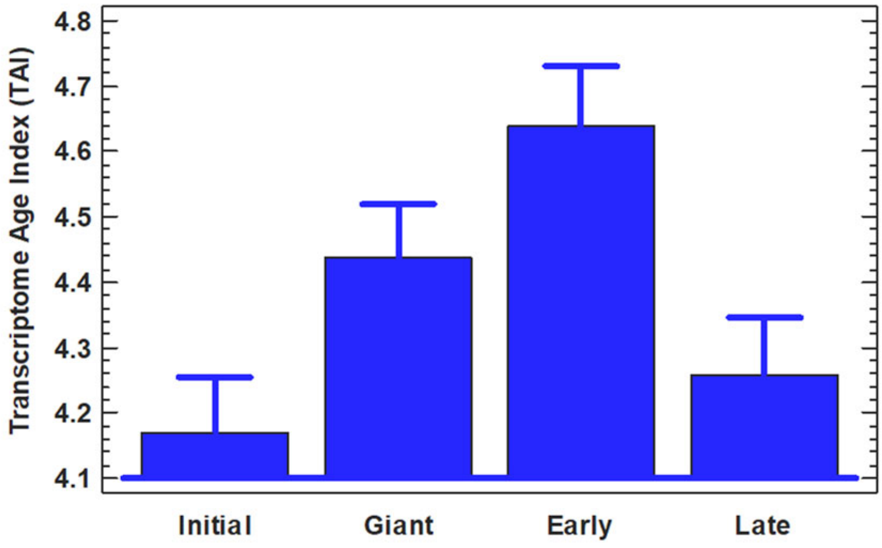

Suppl. Fig. 8. The Transcriptome Age Index (TAI) for all four cell types (initial cancer cells, PGCC, their early and later progeny) (from GSE196453). The higher the TAI, the more recent is the transcriptome.

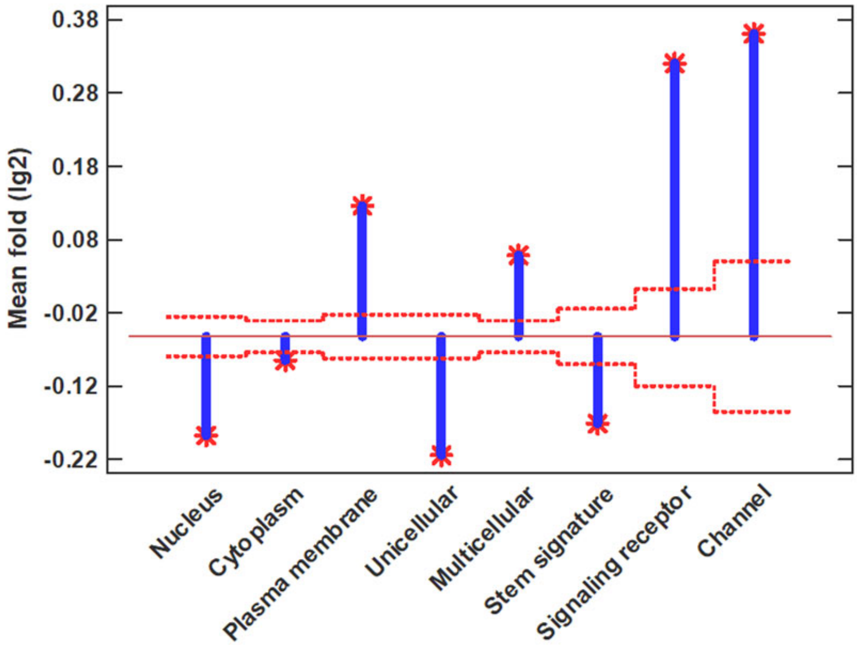

Suppl. Fig. 9. PGCC from prostate cancer cell line (PPC1), compared to initial PPC1 cells (from GSE195919). Gene expression folds for different signatures, with cell cycle genes excluded.

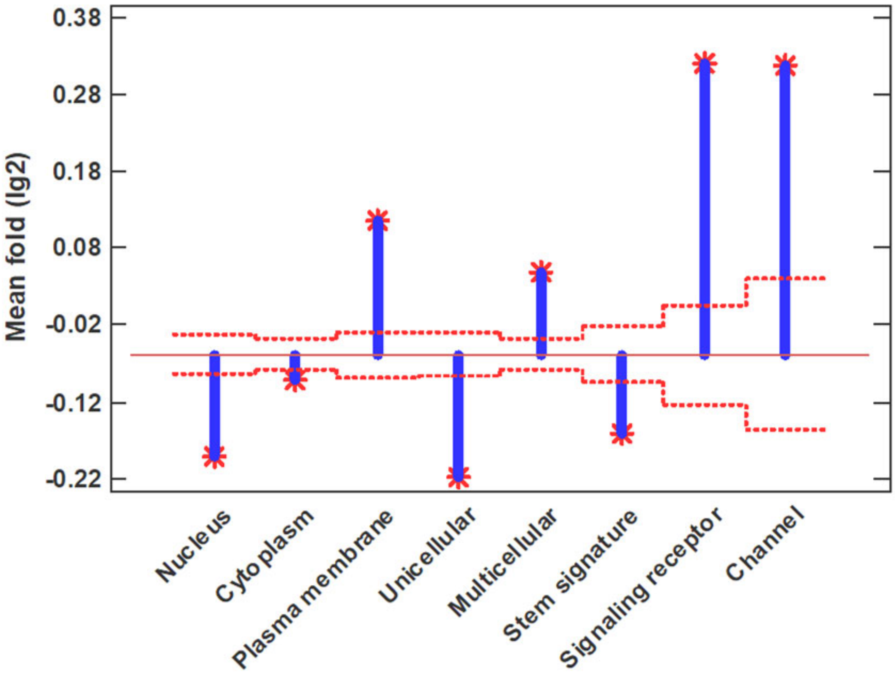

Suppl. Fig. 10. PGCC from prostate cancer cell line (PPC1) treated with pro-drug LCL521, compared to initial PPC1 cells (from GSE195919). Gene expression folds for different signatures, with cell cycle genes excluded.

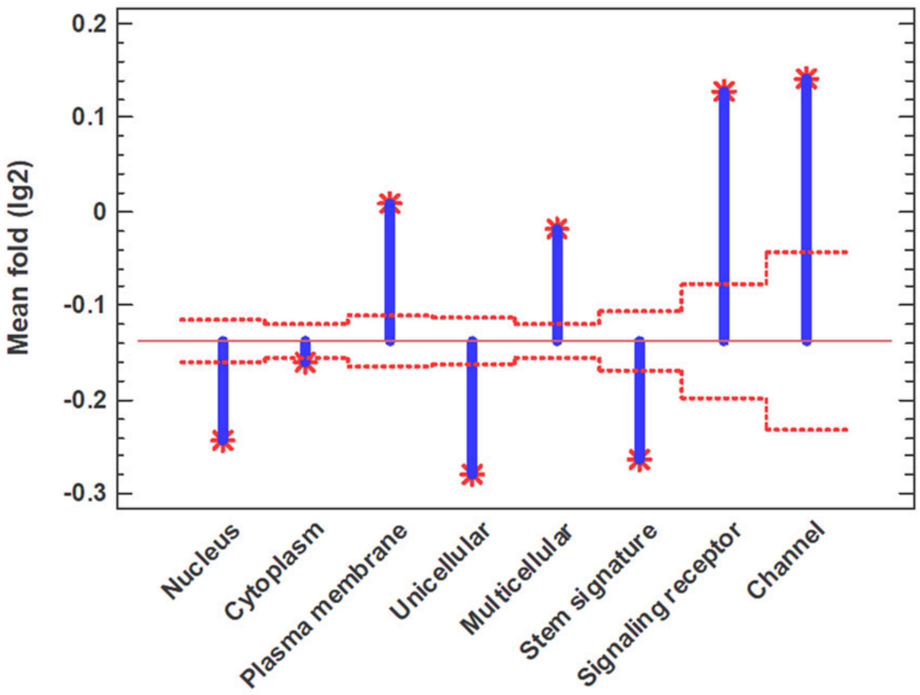

Suppl. Fig. 11. PGCC from ovarian cancer cell line (Hey), compared to initial Hey cells (from GSE178745). PGCC were induced by paclitaxel. Gene expression folds for different signatures, with cell cycle genes excluded.

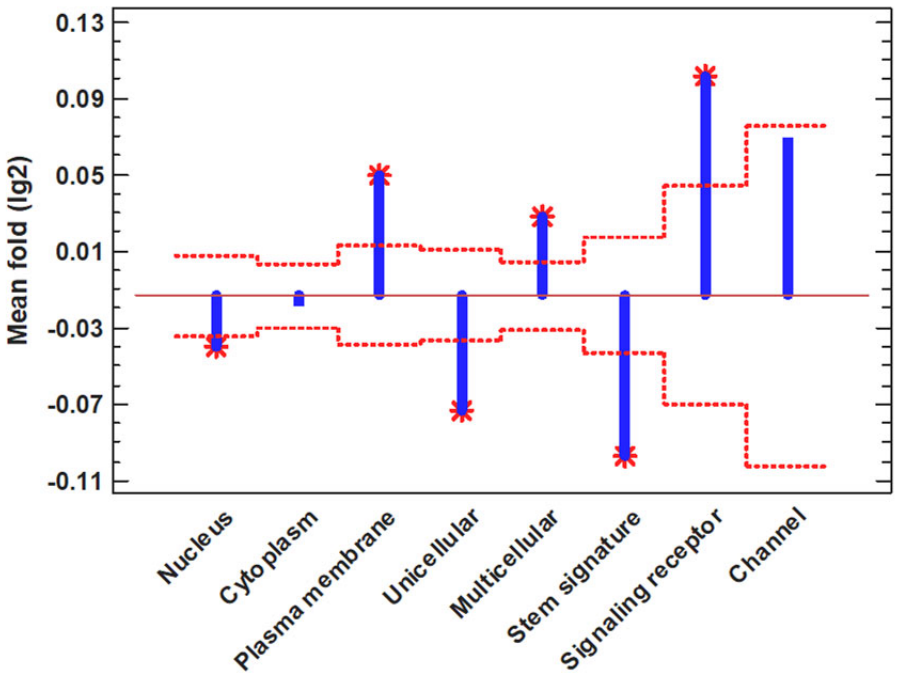

Suppl. Fig. 12. PGCC from ovarian cancer cell line (SKOV3), compared to initial SKOV3 cells (from GSE178745). PGCC were induced by paclitaxel. Gene expression folds for different signatures, with cell cycle genes excluded.

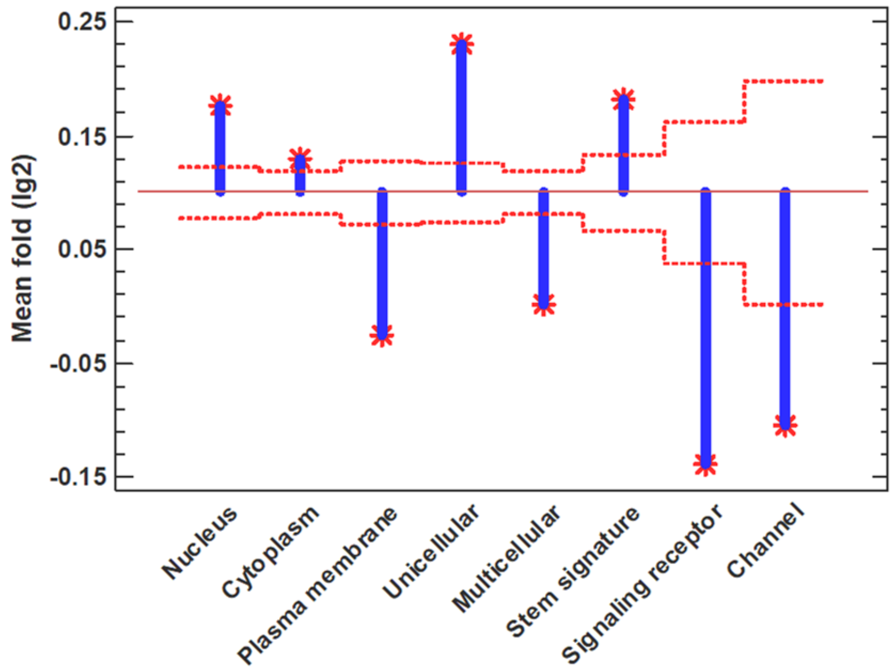

Suppl. Fig. 13. The progeny of PGCC from ovarian cancer cell line (Hey), compared to initial Hey cells (from GSE178745). PGCC were induced by paclitaxel. Gene expression folds for different signatures, with cell cycle genes excluded.

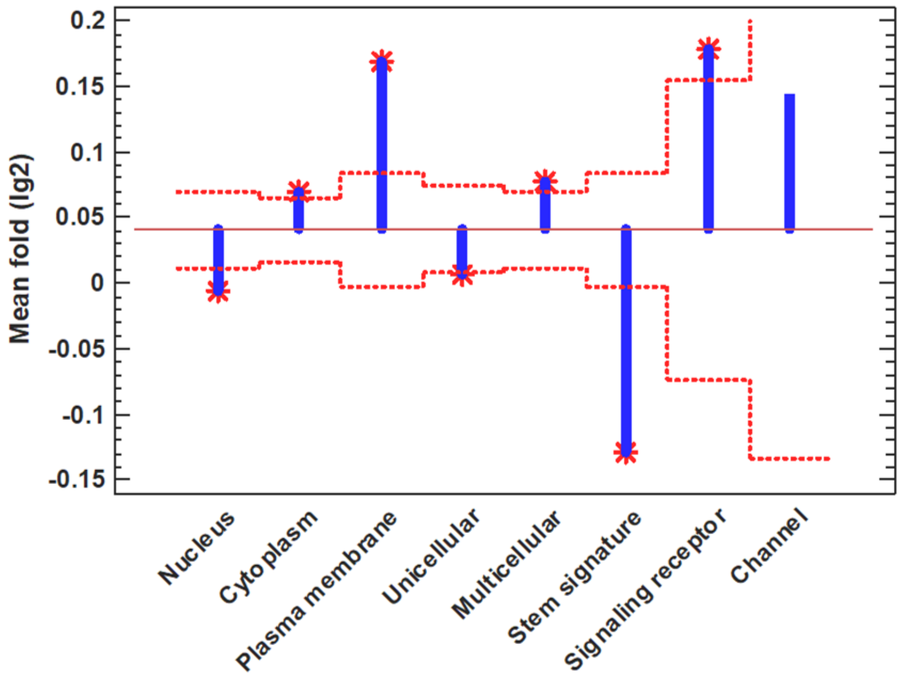

Suppl. Fig. 14. PGCC from ovarian cancer cell line (Hey), compared to initial Hey cells (from GSE229119). PGCC were induced by olaparib. Gene expression folds for different signatures, with cell cycle genes excluded.

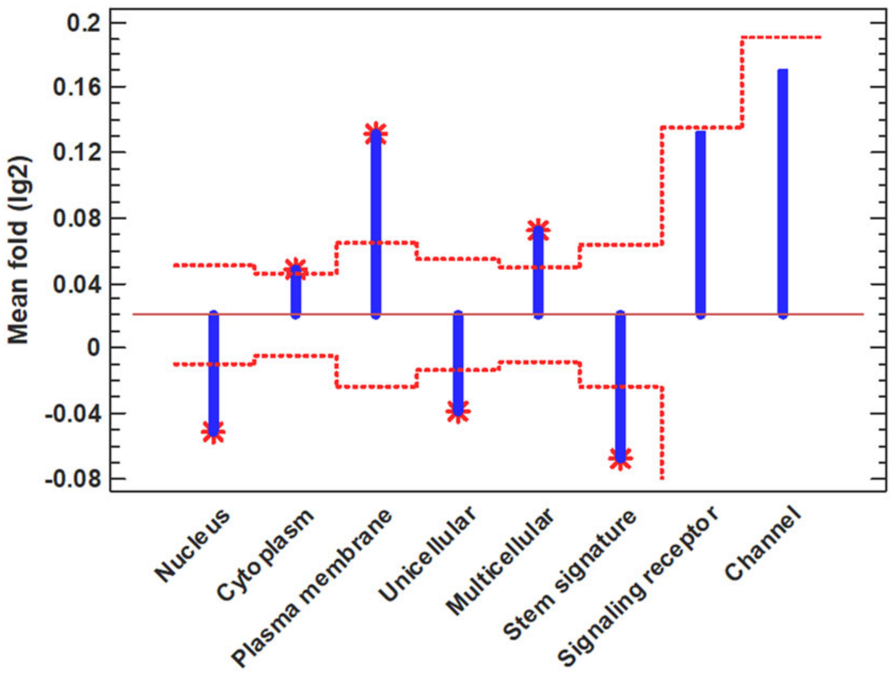

Suppl. Fig. 15. PGCC from breast cancer cell line (MCF7), compared to initial MCF7 cells (from GSE229119). PGCC were induced by olaparib. Gene expression folds for different signatures, with cell cycle genes excluded.

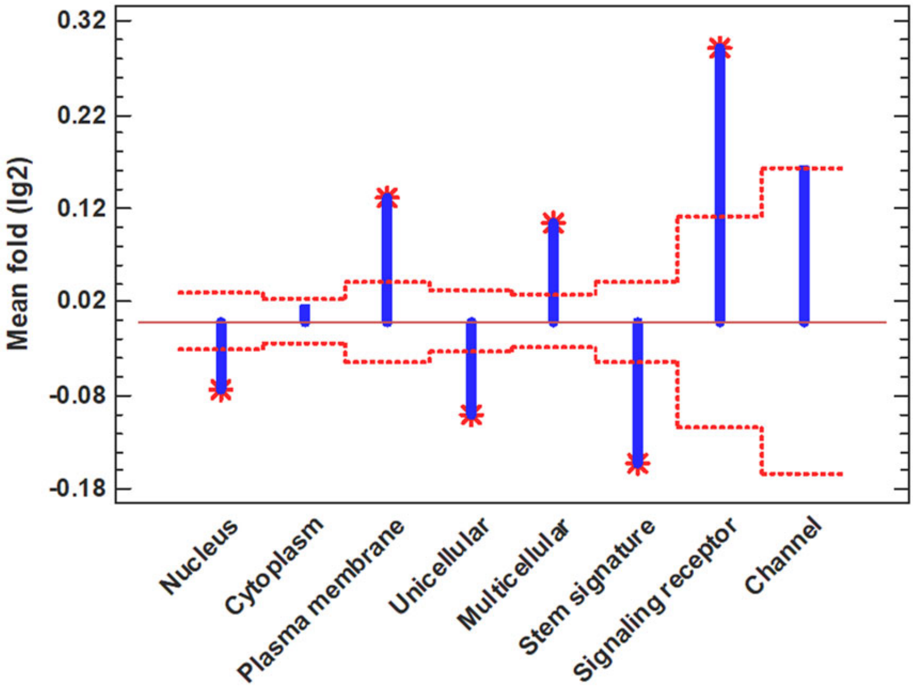

Suppl. Fig. 16. PGCC from primary cell line obtained from individual human high-grade serous ovarian cancers and cultivated in patient-derived xenografts (Org2414), compared to initial Org2414 cells (from GSE229119). PGCC were induced by olaparib. Gene expression folds for different signatures, with cell cycle genes excluded.

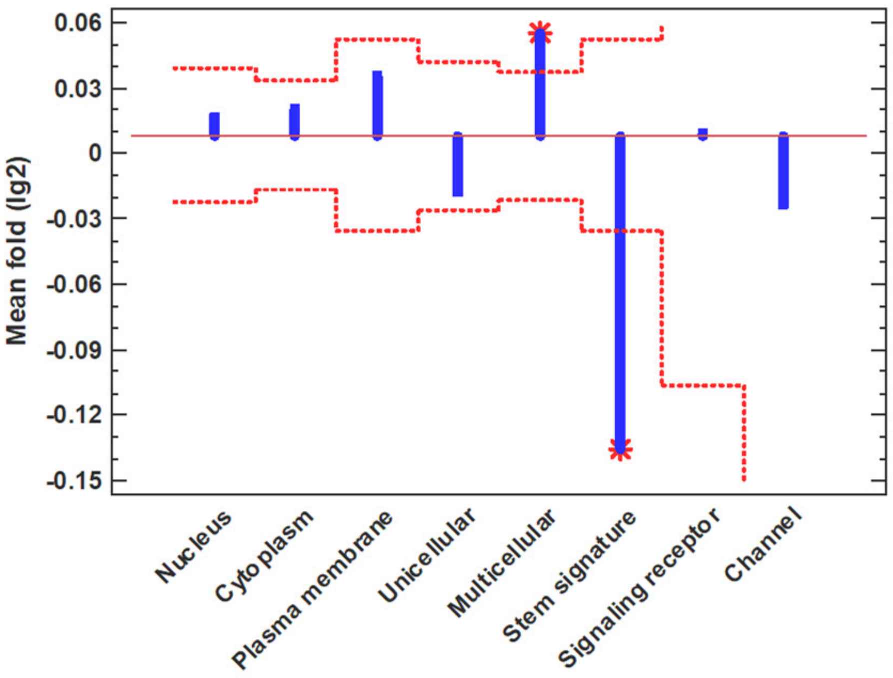

Suppl. Fig. 17. PGCC from ovarian serous adenocarcinoma cell line (Ovca432), compared to initial Ovca432 cells (from GSE229119). PGCC were induced by olaparib. Gene expression folds for different signatures, with cell cycle genes excluded.

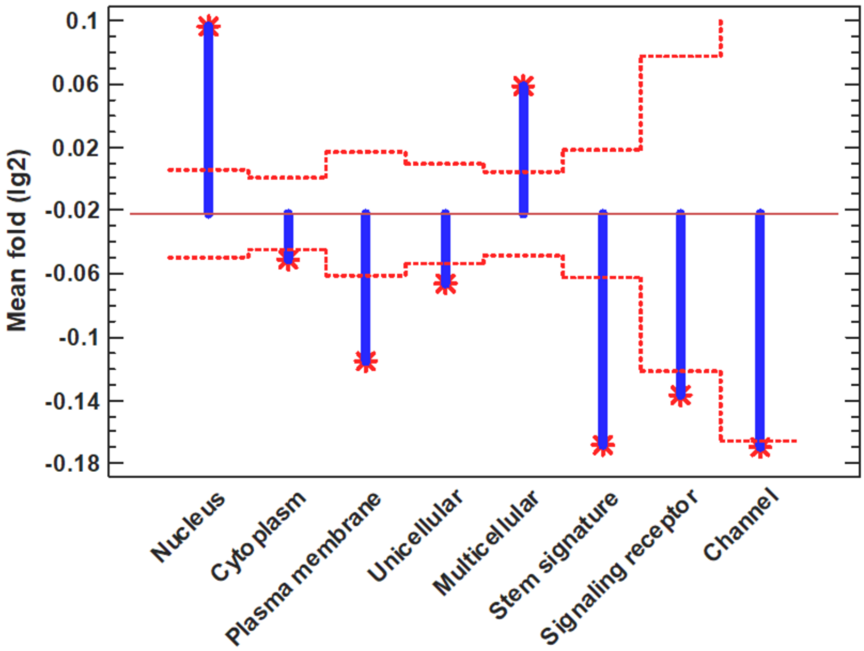

Suppl. Fig. 18. PGCC from primary cell line obtained from individual human high-grade serous ovarian cancers and cultivated in patient-derived xenografts (Org3008), compared to initial Org3008 cells (from GSE229119). PGCC were induced by olaparib. Gene expression folds for different signatures, with cell cycle genes excluded.

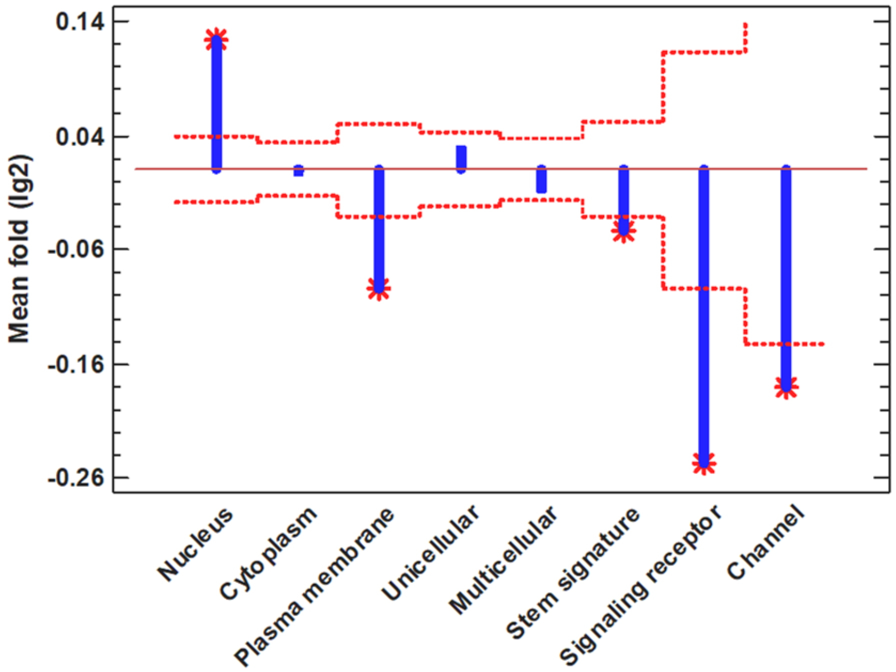

Suppl. Fig. 19. PGCC from primary cell line obtained from individual human high-grade serous ovarian cancers and cultivated in patient-derived xenografts (Org2445), compared to initial Org2445 cells (from GSE229119). PGCC were induced by olaparib. Gene expression folds for different signatures, with cell cycle genes excluded.

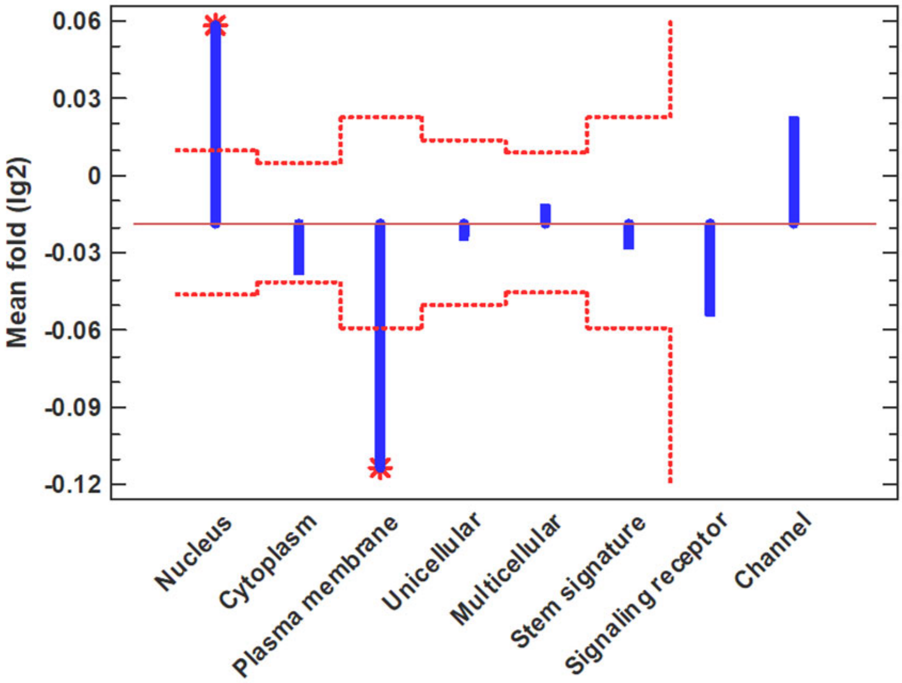

Suppl. Fig. 20. PGCC from ovarian serous adenocarcinoma cell line (SKOV3), compared to initial SKOV3 cells (from GSE229119). PGCC were induced by olaparib. Gene expression folds for different signatures, with cell cycle genes excluded.

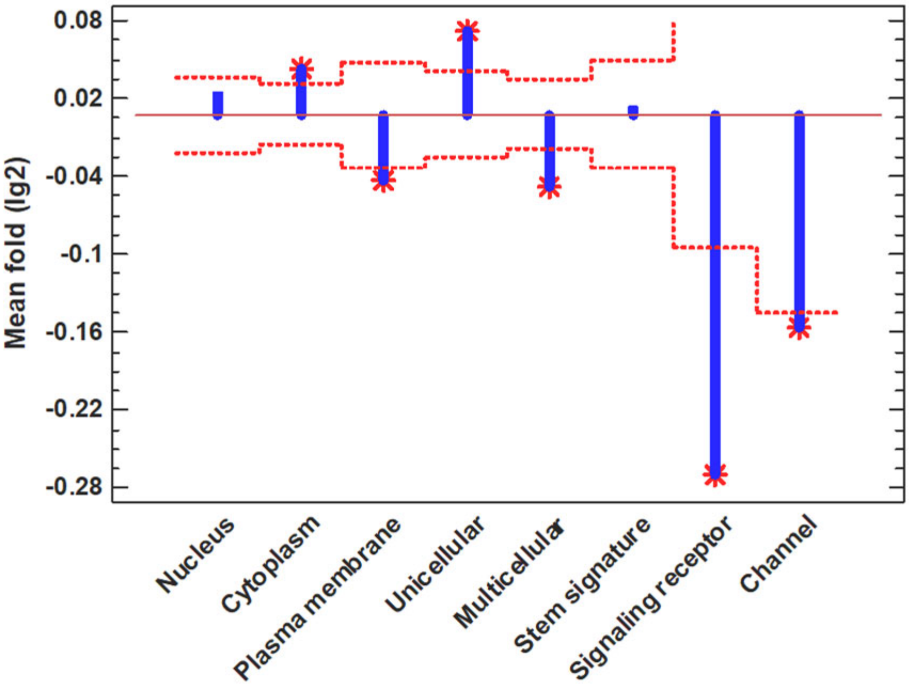

Suppl. Fig. 21. The progeny of PGCC from primary cell line obtained from individual human high-grade serous ovarian cancers and cultivated in patient-derived xenografts (Org2445), compared to initial Org2445 cells (from GSE229119). PGCC were induced by olaparib. Gene expression folds for different signatures, with cell cycle genes excluded.

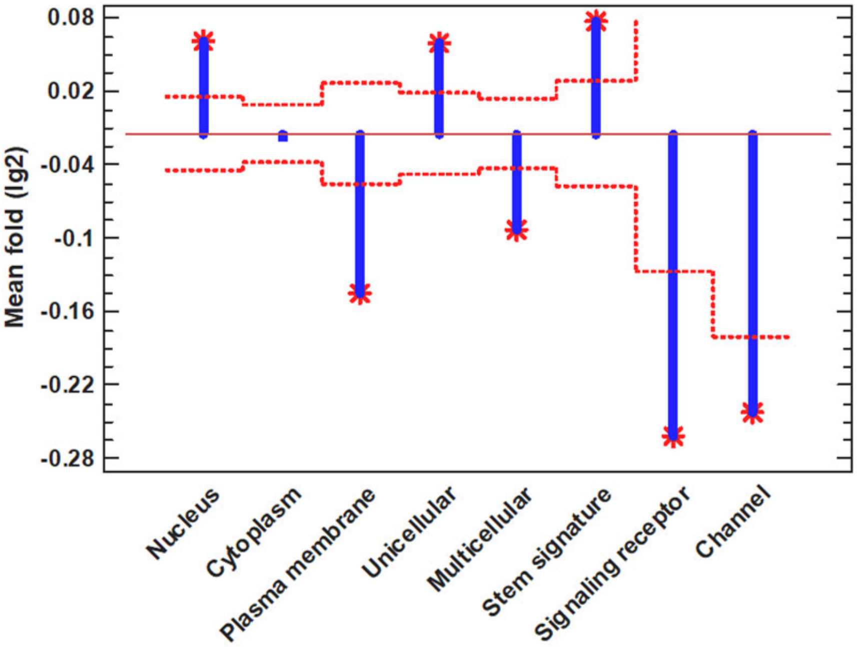

Suppl. Fig. 22. The progeny of PGCC from ovarian serous adenocarcinoma cell line (SKOV3), compared to initial SKOV3 cells (from GSE229119). PGCC were induced by olaparib. Gene expression folds for different signatures, with cell cycle genes excluded.

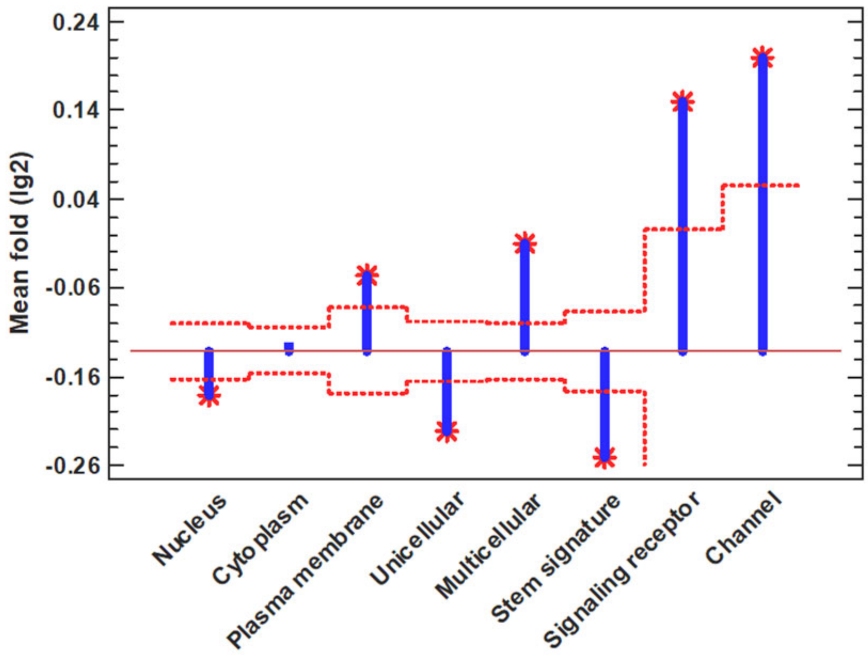

Suppl. Fig. 23. PGCC from breast cancer cell line (MDA), compared to initial MDA cells (from GSE248717). PGCC were induced by docetaxel. Gene expression folds for different signatures, with cell cycle genes excluded.

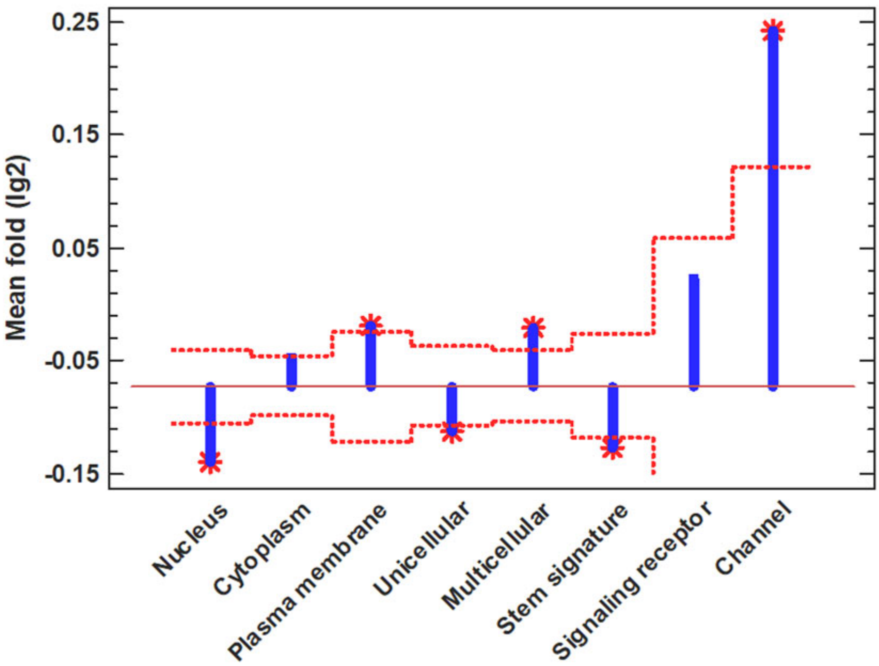

Suppl. Fig. 24. PGCC from breast cancer cell line (SUM159), compared to initial SUM159 cells (from GSE248717). PGCC were induced by docetaxel. Gene expression folds for different signatures, with cell cycle genes excluded.

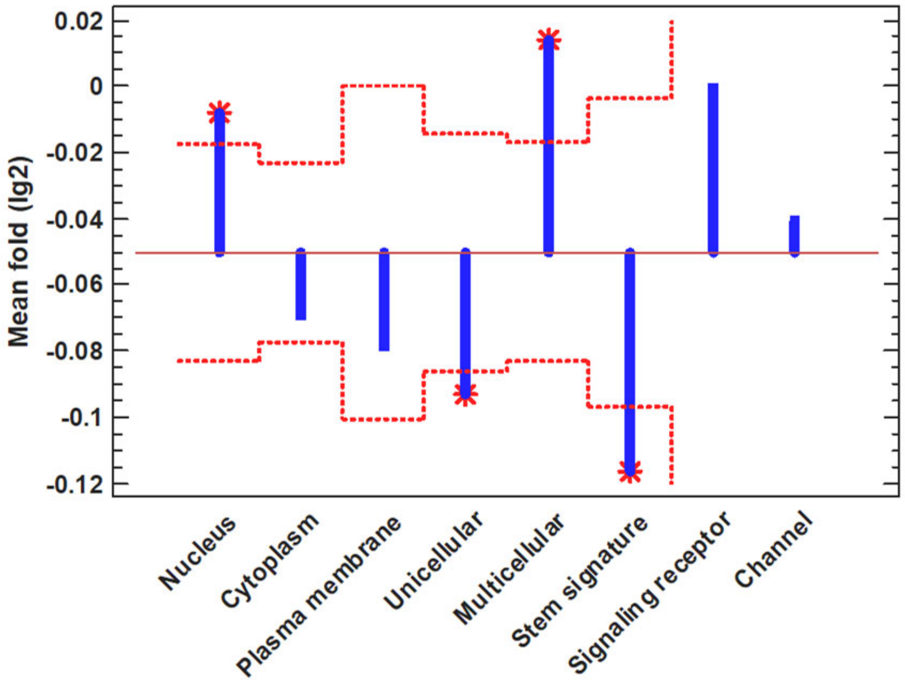

Suppl. Fig. 25. PGCC from breast cancer cell line (Vari068), compared to initial Vari068 cells (from GSE248717). PGCC were induced by docetaxel. Gene expression folds for different signatures, with cell cycle genes excluded.
